# A novel nanoluciferase transgenic reporter to measure proteinuria in zebrafish

**DOI:** 10.1016/j.kint.2022.05.019

**Published:** 2022-06-15

**Authors:** Richard W. Naylor, Emmanuel Lemarie, Anthony Jackson-Crawford, J. Bernard Davenport, Aleksandr Mironov, Martin Lowe, Rachel Lennon

**Affiliations:** 1 Wellcome Centre for Cell-Matrix Research, Division of Cell-Matrix Biology and Regenerative Medicine, School of Biological Sciences, Faculty of Biology Medicine and Health, The University of Manchester, Manchester Academic Health Science Centre, Manchester, UK; 2 Division of Molecular and Cellular Function, School of Biological Sciences, Faculty of Biology, Medicine, and Health, Manchester Academic Health Science Centre, University of Manchester, Manchester, UK; 3 Department of Paediatric Nephrology, Royal Manchester Children’s Hospital, Manchester University Hospitals NHS Foundation Trust, Manchester Academic Health Science Centre, Manchester, UK; 4 EM Core Facility (RRID: SCR_021147), Faculty of Biology, Medicine and Health, University of Manchester, Manchester, UK; 5 Department of Blood Sciences, Grange University Hospital, Llanyravon, Gwent, NP44 8YN, UK

**Keywords:** Zebrafish, proteinuria, Alport syndrome, glomerulus, proximal tubule, basement membrane, type IV collagen

## Abstract

The zebrafish is an important animal system for modelling human diseases. This includes kidney dysfunction as the embryonic kidney (pronephros) shares considerable molecular and morphological homology with the human nephron. A key clinical indicator of kidney disease is proteinuria, but a high-throughput readout of proteinuria in the zebrafish is lacking. We used the Tol2 transposon system to generate a transgenic zebrafish line that uses the *fabp10a* liver-specific promoter to over-express a nanoluciferase molecule fused with the D3 domain of Receptor-Associated-Protein (which we term NL-D3). Using a luminometer, we quantified proteinuria in *NL-D3* zebrafish larvae by measuring the intensity of luminescence in the embryo medium. In the healthy state, NL-D3 is not excreted, but when embryos were treated with chemicals that affected either proximal tubular reabsorption (cisplatin, gentamicin) or glomerular filtration (angiotensin II, Hanks Balanced Salt Solution, Bovine Serum Albumin), NL-D3 is detected in fish medium. Similarly, depletion of several gene products associated with kidney disease (*nphs1*, *nphs2*, *lrp2a*, *ocrl*, *col4a3*, and *col4a4*) also induced NL-D3 proteinuria. Treating *col4a4* depleted zebrafish larvae (a model of Alport syndrome) with captopril reduced proteinuria in this system. Our findings confirm the use of the *NL-D3* transgenic zebrafish as a robust and quantifiable proteinuria reporter. Given the feasibility of high-throughput assays in zebrafish, this novel reporter will permit screening for drugs that ameliorate proteinuria, thereby prioritising candidates for further translational studies.

## Introduction

Proteinuria is a key clinical indicator of kidney disease ^1^. Using urine dipsticks, the presence of protein in the urine can be determined in seconds. Further evaluation of the molecular size of proteins in the urine indicates the site of functional deficit along the nephron; the presence of large proteins in the urine suggests dysfunction in the glomerular filter, whereas the presence of low molecular weight proteins indicates proximal tubule dysfunction ^2,3^. The zebrafish is a popular model organism for the study of kidney development and disease ^4–6^, but a quantitative reporter of proteinuria is currently lacking.

Embryonic zebrafish develop a functioning pronephric kidney that has molecular and structural analogy to a human nephron ^7^. The pronephros consists of a single midline fused glomerulus attached to bilateral tubules that are segmented along their anterior-posterior axis into distinct proximal and distal domains ^7,8^. Until recently, the best method of determining kidney function in zebrafish embryos was the use of fluorescent dextrans of varying molecular weights that distinguish between glomerular and proximal tubule dysfunction ^9^. However, fluorescent dextran is not specific to the cellular machinery of protein endocytosis, it is passively internalised in the fluid bulk. As such, these methods are useful, but they are not quantitative, do not specifically identify megalin-mediated endocytosis defects in the proximal tubule, and cannot be used in a high-throughput manner.

Recently, a zebrafish transgenic reporter (*fabp10a:½vdbp-mCherry)* has enabled embryo medium to be collected and assayed for low molecular weight proteins ^10^. This powerful system permits quantification of proteinuria as a readout of dysfunction of the lrp2/megalin endocytosis pathway in the proximal tubule. It reports proximal tubular dysfunction following downstream processing with immunofluorescence imaging or ELISA assays to quantify protein uptake in the proximal tubules and proteinuria. This genetic tool highlights the potential of transgenics to develop reporters of proteinuria in the zebrafish.

Here, we describe a novel nanoluciferase based reporter of proteinuria in zebrafish. Excreted nanoluciferase protein in the embryo medium is detected by luminescence following simple addition of its substrate luciferin. With this new system, perturbations in both proximal tubular and glomerular function can be assayed, and its simple and easy application makes it amenable to use in a high-throughput manner.

## Short Methods

### Zebrafish husbandry

Zebrafish were maintained and staged according to established protocols ^11^ and in accordance with the project licenses of Martin Lowe (70/9091) and Rachel Lennon (P1AE9A736) under the current guidelines of the UK Animals Act 1986.

### Zebrafish injections and treatments

Embryos were injected at the stage indicated in the text and were treated with drugs/fluorescent tracers by either incubation in embryo medium (after dechorionation) or by microinjection into the common cardinal vein. For morpholino treatments, final concentrations of 0.15 mM (*nphs1*^MOex25^) and 0.25 mM (*nphs2*^MOex3^) were used. For *lrp2a* and *ocrl* knockdowns, a *p53* morpholino was also co-injected at the same concentration (0.24 mM). CRISPR-Cas9 depletion is described in ref^12^. gRNAs were re-suspended in nuclease-free water to a concentration of 20 μM. For the injection mix, 4 μM of each gRNA was combined with Cas9 (NEB #M0646) and Cas9 buffer.

### Zebrafish proteinuria reporter assay

Embryos were grown to 4 days post fertilisation (dpf), then three embryos per well were placed in one well of a 96-well dish. E3 embryo medium was removed and replaced with 200 μl fresh E3. 24 hours later 50 μl of E3 medium was removed from each well and placed in the corresponding well of a fresh opaque 96-well plate. 50 μl of substrate from the NanoGlo® Luciferase Assay System (Promega #N1110) was then added to each well. Plates were then briefly spun down at 700 rpm for 1 minute and then immediately assayed for luminescence on a Flexstation 3 multi-mode microplate reader.

### In situ hybridisation

Whole-mount in situ hybridisation on zebrafish embryos was performed as previously described ^13^. Digoxigenin-labelled anti-sense riboprobes were made using T3 RNA polymerase transcription kits (Roche Diagnostics). Probe templates for zebrafish *col4a3, col4a4* and *col4a5* were generated by PCR amplification from cDNA taken from 4 dpf zebrafish embryos. Whole embryos were imaged on a Leica M205 FA upright stereofluorescence microscope, and transverse sections were imaged on an Olympus BX63 snapshot slide scanner microscope.

### Cryosectioning and immunofluorescence

4 dpf zebrafish embryos were fixed in Dent’s fixative (80% Methanol, 20% DMSO) or 4% PFA (for podocin staining) overnight at 4°C. These embryos were then flash frozen in cold isopentane after embedding in OCT. Samples were then sectioned on a Leica CM1950 cryostat and processed for antibody staining. The primary antibodies used were NPHS2 (Abcam #ab50339; 1:250) and pan collagen IV (Abcam #ab6586; 1:250). Images were collected on a Leica TCS SP8 AOBS inverted confocal using a 60X Plan Fluotar objective. The confocal settings were as follows, pinhole 1 airy unit, scan speed 1000Hz unidirectional, format 1024 x 1024. Images were collected using the white light laser with 488nm (10%) laser lines.

### Transmission electron microscopy

Samples were prepared according to protocols described previously ^14^. Images were taken on T12 Biotwin transmission electron microscope. Distances were measured in Fiji/ImageJ, measurements for foot process width were taken and normalized to the length of the glomerular basement membrane. The total number of GBM width measurements for each sample was: *control* crispant, n=36; *col4a3* crispant, n=32; *col4a4* crispant, n=37; *col4a5* crispant, n=30. For foot process/ GBM length measurements, five TEM images per treatment were used and the total GBM length (nm) was measured. The number of foot processes along this GBM length was counted, then this number was divided by the GBM length. The mean ± SEM was calculated, and a student’s t-test was performed using GraphPad Prism version 8.4.3 for 120 Windows, GraphPad Software, San Diego, California 121 USA,www.graphpad.com

## Results

### Recombinant NL-D3 can be endocytosed in vitro

**Figure 1:**
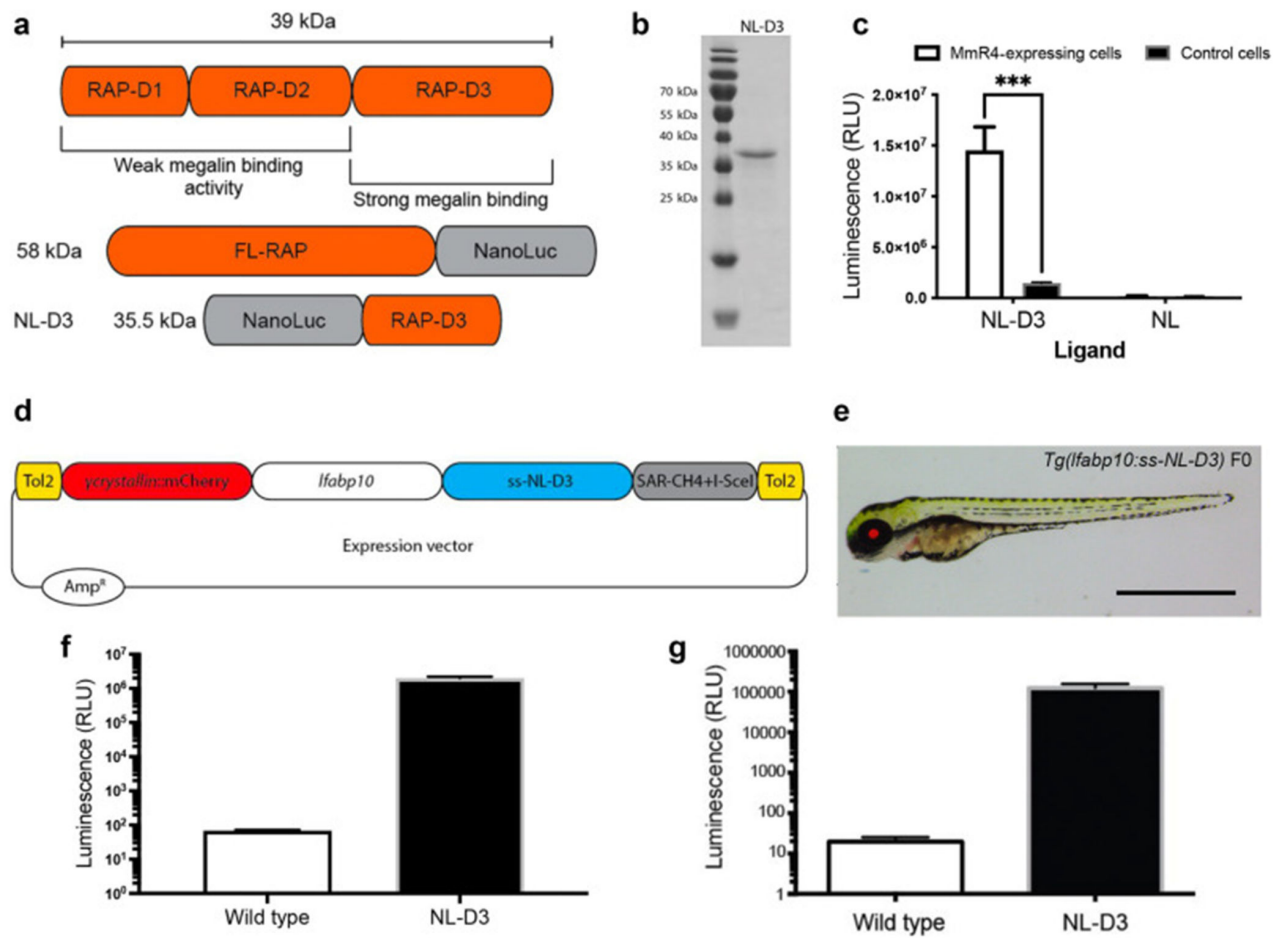
NL-D3 is uptaken by the megalin endocytosis pathway A) Top schematic showing binding affinity to megalin of the three D-domains of the Receptor Associated Protein (RAP). Bottom schematics show the size and orientation of full-length RAP bound to the N-terminus of Nano-Luc and the RAP D3 domain bound to the C-terminus of Nano-Luc (NL-D3, which was used in this work). B) SDS-PAGE gel showing the recombinantly expressed and purified NL-D3 protein alongside molecular weight markers. C) Graph showing the internalisation of NL-D3 or untagged NL into MmR4 mini-megalin expressing cells and control non-megalin expressing cells. D) Schematic for the *y-crystallin:mcherry/fabp10a:NL-D3* Tol2 vector used to generate transgenic zebrafish. E) Panel shows lateral view of a 5 dpf *NL-D3* transgenic zebrafish embryo under excitation for red fluorescence to highlight the mCh expression in the lens. Scale bar = 1 mm. F) Histogram showing relative luminescence units (RLU) in whole embryo lysates of wild-type and *NL-D3* 5 dpf embryos. G) Logarithmic histogram showing the RLU of 1 μl blood from adult wild-type and *NL-D3* zebrafish.

We aimed to develop a transgenic reporter to study endocytosis in the zebrafish pronephric proximal tubule. An important component of endocytic pathways in the proximal tubule is the giant 600 kDa megalin receptor, which has multiple ligands ^15^. Megalin function and processing is dependent on Receptor-Associated-Protein (RAP) ^16^, which contains three D-domains that bind with varying affinity to megalin ^17^ (Figure 1A). For our reporter protein, we fused rat RAP to nanoluciferase (NL). A full-length RAP-NL fusion yields a protein that is 58 kDa in size (Figure 1A). We hypothesized that limiting the size of the reporter protein would aid its transit through the glomerular capillary wall and so we fused the 14 kDa D3 domain of RAP to the C-terminus of NL to create NL-D3, which has a predicted size of 35.5 kDa (Figure 1A). We first wanted to confirm that the NL-D3 fusion protein could report megalin-dependent endocytosis. For this purpose, we expressed recombinant NL-D3 in *E. coli* and used affinity isolation to purify the protein (Figure 1B). The recombinant NL-D3 was then used in uptake experiments on HEK293 cells stably expressing a truncated version of megalin (containing the cytoplasmic tail, transmembrane domain and the fourth of four large extracellular repeats, named MmR4). Uptake was measured by lysing the cells and measuring luminescence and this showed that the HEK293-MmR4 cells incubated with recombinant NL-D3 had ~10-fold higher levels of uptake compared to control HEK293 cells incubated with recombinant NL-D3 (Figure 1C). Neither control HEK293 cells nor HEK293-MmR4 cells displayed luminescence after incubation with NL (Figure 1C). These *in vitro* results indicate that NL-D3 is bound by megalin and readily endocytosed.

### Development of y-crystallin:mcherry/fabp10a:NL-D3 transgenic zebrafish

To develop a viable *in vivo* reporter of proximal tubule endocytosis we turned to the zebrafish model. We used the versatile Tol2 gene transfer system ^18^ to generate transgenic zebrafish that express NL-D3 under the control of the liver-specific *fabp10a* promoter (Figure 1D). To enable selection, the transposable element dually expressed mCherry under the control of the *y*-crystallin promoter (Figure 1E). Whole embryo lysates from stably transgenic 5 dpf *y*-crystallin:mcherry/fabp10a:NL-D3 zebrafish (hereon in termed *NL-D3*) had ~100,000-fold more luminescence than wild-type embryos (Figure 1F). A similar increase in luminescence was observed in blood isolated from adult-stage *NL-D3* reporter fish (Figure 1G). We also measured the amount of NL-D3 in adult organ tissues, with the most luminescence observed in the liver (Figure S1). These results indicate liver-specific expression of NL-D3 in transgenic zebrafish results in the robust release of this reporter into the blood vasculature. We have bred multiple generations of *NL-D3* transgenic zebrafish and have not observed any impact on overall fish health or life longevity. Histological analysis of livers from *NL-D3* zebrafish and age-matched controls showed no overt changes (Figure S2).

### Proteinuria is detected in NL-D3 embryos with proximal tubule dysfunction

**Figure 2:**
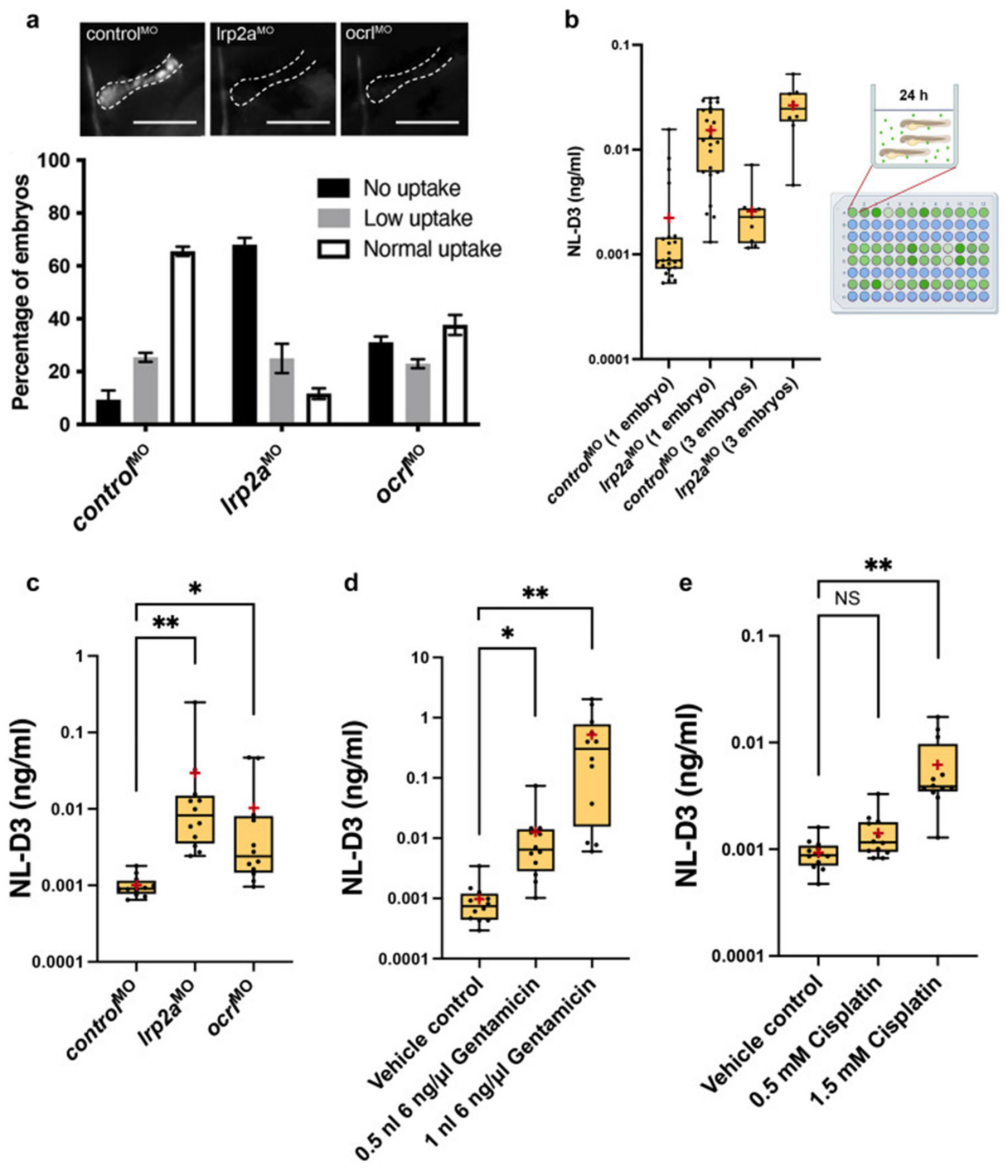
*NL-D3* zebrafish can be used to assay proximal tubule dysfunction A) Above panels show close-up lateral views of pronephric proximal tubules after indicated treatments and 1-2 hours post dextran injection. Scale bars = 100 μm. Below bar chart showing the level of uptake of a 10 kDa fluorescent dextran in *control* (n=40), *lrp2a* (n=39), and *ocrl* (n=42) morphant zebrafish embryos B) Box and whisker plot showing NL-D3 levels detected in controls and *lrp2a*morphants incubated as one embryo per well (n=24) or 3 embryos per well (n=9). Schematic showing the experimental setup for the *NL-D3* zebrafish embryos, each 96-well plate was assessed for luminescence on a luminometer. C) Box and whisker plot showing the amount of NL-D3 detected in the embryo medium in *control* (n=12), *lrp2a* (n=12) and *ocrl* (n=12) morphants. D) Box and whisker plot showing the amount of NL-D3 detected in the embryo medium in vehicle control (DMSO) and two volumes of injected gentamicin (n=12 for each assay). E) Box and whisker plot showing the amount of NL-D3 detected in the embryo medium in control and two concentrations of cisplatin (n=12 for each assay). For box and whisker plots in B)-E), median is shown as a line and mean is shown as a red cross-hair.

To test if NL-D3 passes through the glomerular filter and is re-absorbed by the megalin endocytic pathway in the pronephric proximal tubule, we knocked down *lrp2a* (the gene that encodes for megalin) using a previously described morpholino oligonucleotide ^19^. Reuptake of a 10 kDa fluorescent dextran in proximal tubules was scored as normal, low or none (Figure S3). Depletion of *lrp2a* was sufficient to severely reduce uptake of a 10 kDa fluorescent dextran in proximal tubules (n=39) when compared to controls (n=40) (Figure 2A), confirming these morphant embryos have perturbed proximal tubule reabsorption. For the NL-D3 proteinuria assay, *lrp2a* morphants at 4 dpf were placed in one-well of a 96-well plate, the embryo medium was replaced, and embryos were cultured further before NL-D3 content in the embryo medium was measured on a luminometer. We found that three embryos per well (n=9 wells) yielded less variability in the luminescence measurements compared to one embryo per well (n=24) (Figure 2B). We also measured luminescence of embryo medium taken from *lrp2a* morphants after 1 hour, 4 hours, 8 hours, and 24 hours (n=9 for each time-point). This showed that the NL-D3 reporter is sensitive enough to assess proteinuria after as little as one hour, but the separation between controls and experimental samples was greatest at 24 hours (Figure S4), thus we used this time-point in all our assays. Relative luminescence units (RLUs) obtained from the luminometer were quantifiably converted to NL-D3 amount (ng/ml) using a standard curve generated from recombinant NL-D3 (Figure S5). Depletion of *lrp2a* caused a ~29-fold increase in NL-D3 present in the embryo medium (n=12, Figure 2C). Another gene product involved in proximal tubule endocytosis is *OCRL*, and pathogenic variants cause Lowe syndrome, which is associated with low molecular weight proteinuria ^20,21^. Morpholino knockdown of the zebrafish ortholog *ocrl* using a previously validated morpholino ^22,23^ caused a reduction in 10 kDa fluorescent dextran uptake in the proximal tubule (n=42, Figure 2A), and induced NL-D3 proteinuria (~10-fold increase, n=12, Figure 2C).

We also tested the effect of nephrotoxins that target proximal tubules and induce proteinuria. Gentamicin and cisplatin are two drugs that are well established nephrotoxins that cause kidney damage primarily by proximal tubule cell death and they have previously been used to ablate the zebrafish proximal tubule epithelium ^24^. Analysis of NL-D3 secretion into embryo medium showed that 0.5 nl of 6 ng/μl gentamicin increased proteinuria by ~13-fold compared to vehicle controls (n=12, Figure 2D). Treating embryos with double the amount of gentamicin further increased the proteinuria to ~534-fold over controls (n=12, Figure 2D), suggesting a dose response is detectable in the *NL-D3* reporter. A similar dose-response was obtained with cisplatin, a low (0.5 mM) dose causing a ~1.5-fold increase in proteinuria, and a high (1.5 mM) dose causing a ~6.7-fold increase (n=12, Figure 2E). Taken together, these results confirm the *NL-D3* transgenic fish as a viable tool for detecting proteinuria due to proximal tubular dysfunction after genetic perturbation or pharmacological treatment.

### Proteinuria is detected in NL-D3 embryos with glomerular dysfunction

**Figure 3:**
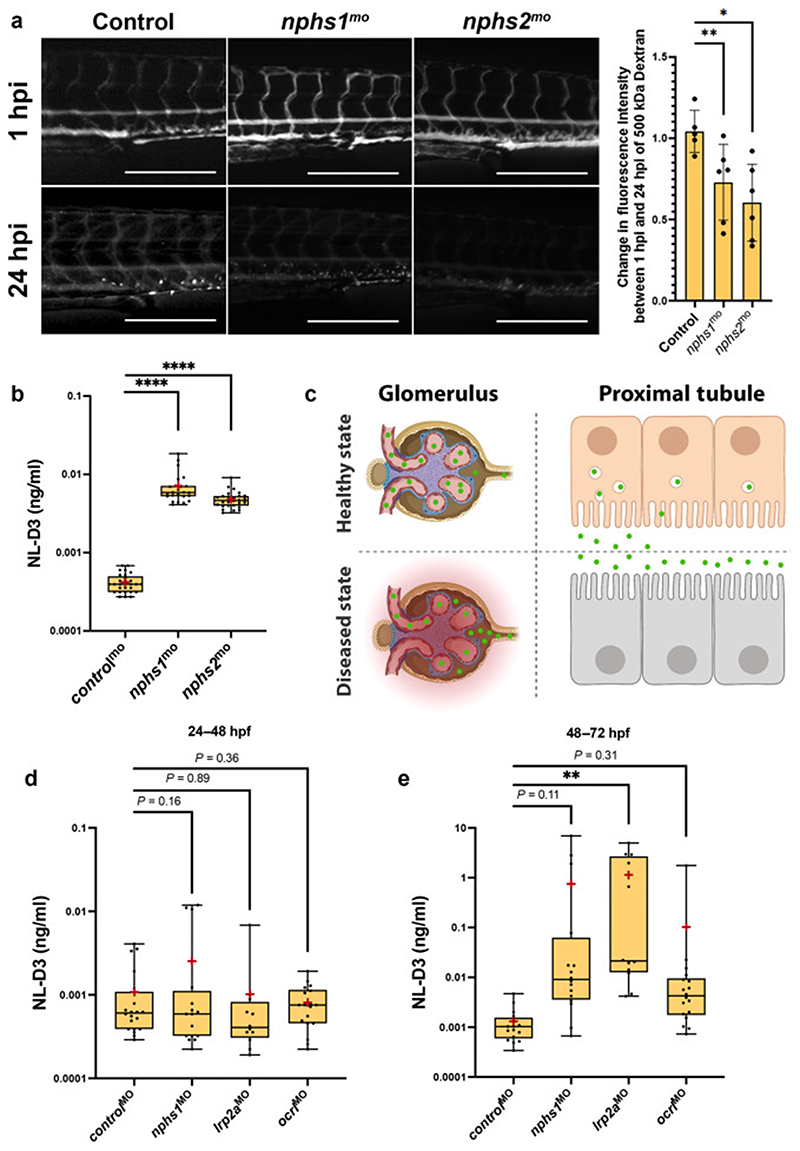
*NL-D3* zebrafish can also be used to study glomerulopathies A) Panels showing 4 dpf embryos injected with a 500 kDa FITC-conjugated dextran at 1 hour post injection (hpi) and 24 hpi after the treatments shown (*control*^mo^ n=5, *nphs1*^mo^ n=6, *nphs2*^mo^ n=6). Scale bar = 250 μm. Bar chart to the right shows the change in fluorescence intensity measured in the dextran-injected embryos at 24 hpi compared to 1 hpi. B) Box and whisker plot showing the amount of NL-D3 detected in the embryo medium in *control* (n=29), *nphs1* (n=28), and *nphs2* (n=22) morphant embryos. C) Schematic highlighting the barrier function of the healthy state glomerular filter to NL-D3 and its re-uptake in healthy state proximal tubules (top panels). The lowered barrier function in the glomerulus and the reduced endocytosis activity in the proximal tubules is displayed in the bottom panels, to schematically represent the dysfunction in these two tissues in a diseased state. D) and E) Box and whisker plots showing NL-D3 detected in embryo medium taken from embryos treated as shown between 24-48 hpf (D) and 48-72 hpf (E) (*control*^mo^ n=20, *nphs1*^mo^ n=16, *lrp2a*^mo^ n=6, *ocrl*^mo^ n=18). In B), D) and F) median is shown as a line and mean is shown as a red cross-hair.

We further analysed the effects of perturbing the expression of glomerular specific genes to determine whether the *NL-D3* reporter could also be used to measure proteinuria associated with glomerular dysfunction. *NPHS1* and *NPHS2* encode for proteins (nephrin and podocin, respectively) that establish the podocyte slit diaphragm. Pathogenic variants in these genes cause nephrotic syndrome, which is characterised by excessive proteinuria. Morpholino-mediated depletion of nephrin and podocin in zebrafish has previously recapitulated phenotypes observed in human patients with disease-causing variants in *NPHS1* and *NPHS2* ^25,26^. Using identical morpholino oligonucleotides to those used by Fukuyo et al (2014), we observed clearance of a 500 kDa FITC-conjugated dextran from the vasculature (*std control*^mo^ n=5, *nphs1*^mo^ n=6, *nphs2*^mo^ n=6, Figure 3A), confirming these morpholinos cause glomerular dysfunction. We also observed increased NL-D3 in the embryo medium analysed from zebrafish morphants depleted of either *nphs1* (~17-fold increase, n=28) or *nphs2* (~11.5-fold increase, n=22) compared to control morphants (n=29) (Figure 3B). These results demonstrate that the *NL-D3* reporter functions as a dual reporter of both glomerular and proximal tubular dysfunction (Figure 3C).

Transmission electron microcsopy analysis of glomerular development in the zebrafish pronephros suggests that a fully formed filtration barrier is established at approximately 4 dpf ^27^. Pronephric tubule differentiation occurs earlier than this, with a brush border and proximal-distal segmentation present by 48 hpf ^7,8,28^. We tested whether the *NL-D3* reporter could be used at earlier stages of development to detect proteinuria associated with proximal tubule dysfunction (*lrp2a* and *ocrl* depletion) or glomerular dysfunction (*nphs1* depletion). We found that neither tubular- or glomerular-associated proteinuria is detectable in the reporter between 24 and 48 hpf in controls (n=20), *nphs1* morphants (n=16), *lrp2a* morphants (n=12), or *ocrl* morphants (n=18) (Figure 3D). Analysis of the same embryos between 48 and 72 hpf identified a significant increase in proteinuria in *lrp2a* morphants, but embryos depleted of *ocrl* or *nphs1* did not show proteinuria (Figure 3E). These results suggest that the *NL-D3* line can report proteinuria associated with severe tubular dysfunction from 48 hpf, but glomerular dysfunction and less severe tubular dysfunction is not detectable before 72 hpf.

### Expression analysis of type IV collagen genes in zebrafish

**Figure 4:**
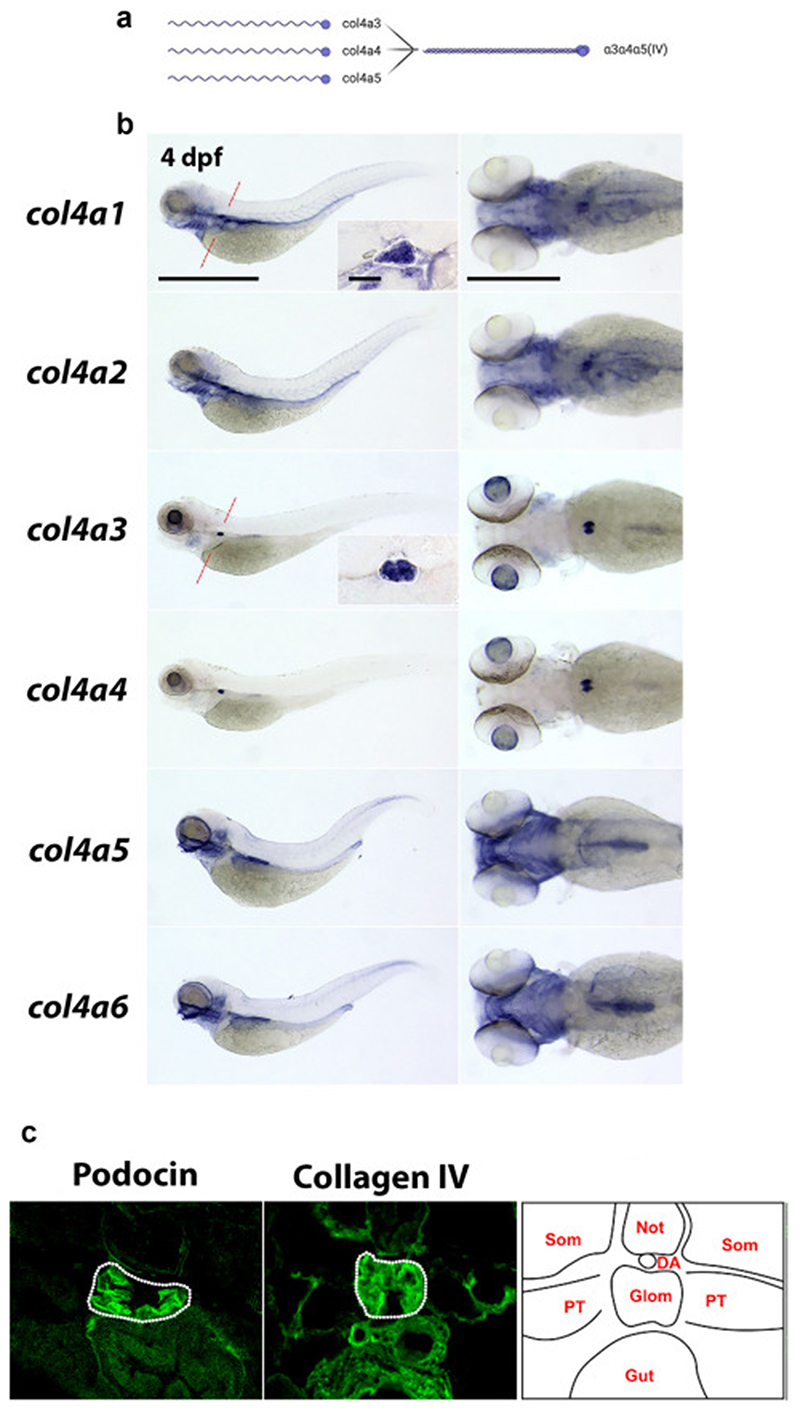
Type IV collagens are expressed in the zebrafish glomerulus A) Schematic (created with Biorender) showing the three type IV collagen alpha chains expressed in the glomerular basement membrane (α3, α4, and α5) on the left, and their interaction to form the α4α4α5(IV) heterotrimer on the right. B) In situ hybridisations showing the expression patterns of all six type IV collagen chains in zebrafish at 4 dpf. Panels on the left are lateral views of the whole embryo (anterior left, scale bar = 100 μm). Panels on the right are dorsal views of the head and anterior trunk region (anterior to the left, scale bar = 50 μm). Inlet panels in the *col4a1* and *col4a3* whole embryo profiles show the glomerulus in transverse section (scale bar = 10 μm) through the point in the embryo highlighted in a red dashed line on the whole embryo image. C) Panels show transverse sections through the glomerulus of 4 dpf zebrafish embryos immunostained for podocin (left panel) and pan-collagen IV (middle panel). Right panel shows schematic representation of the tissues in cross-section at this position.

The potential for the *NL-D3* reporter line to measure glomerular dysfunction is of particular interest given the plethora of kidney diseases associated with glomerular pathology. One such condition is Alport syndrome, which is caused by variants in *COL4A3, COL4A4 or COL4A5* in humans. These genes encode monomers that trimerise to assemble the collagen a3a4a5(IV) network (Figure 4A), which is localized to the mature glomerular basement membrane (GBM). The a3a4a5(IV) network is postulated to provide mechanical strength to the GBM, allowing it to counteract the high intraglomerular forces generated by capillary wall stress ^29–31^. Variants in *COL4A3, COL4A4* or *COL4A5* lead to reduced or absent collagen a3a4a5(IV) in the GBM, affecting its long-term function. Patients with Alport syndrome initially present with microhaematuria, followed by proteinuria and a progressive decline in kidney function leading to kidney failure.

The expression profile of type IV collagen chains in the zebrafish glomerulus has not previously been characterised. Zebrafish have six type IV collagen genes, *col4a1-a6*, which are orthologs of the six type IV collagen genes found in humans (*COL4A1-A6*). We performed *in situ* hybridisation to detect the spatial organisation of transcripts for *col4a1-a6* in 4 dpf zebrafish embryos, a time-point when the glomerulus is fully developed ^27^ (Figure 4B). Expression profiles of *col4a1* and *col4a2* were identical, which is expected given vertebrate type IV collagen gene expression is regulated via putative bidirectional promoters for *col4a1*/*col4a2*, *col4a3*/*col4a4*, and *col4a5*/*col4a6 ^32,33^*. *col4a1* and *col4a2* transcripts were detected in the blood vasculature, most notably in the aortic arches, central arteries, and primordial hindbrain channels in the head region (Figure 4B). Glomerular localisation for *col4a1* was clearly observed in transverse sectioned embryos (Figure 4B, inlet). In these sections, *col4a1* also labelled numerous trunk epithelial structures (gut endoderm, pronephric tubules, dorsal aorta, and posterior cardinal vein). When compared to *col4a1* and *col4a2, col4a3* and *col4a4* expression profiles were more restricted. High levels of expression for *col4a3* and *col4a4* were observed in the lens capsule and the glomerulus, as well as weak detection in the branchial arches (Figure 4B). Expression of *col4a5* and *col4a6* showed strong localisation for transcripts in the craniofacial region, branchial arches, otic vesicle, developing swim bladder, and posterior gut (Figure 4B). Using *in situ* hybridisation we were unable to detect expression of *col4a5* in the glomerulus, despite using different probes. This result is anomalous in the context of type IV collagen chain trimerisation. Biochemical studies suggest the α3(IV) and α4(IV) chain non-collagenous 1 domains can only trimerise with one α5(IV) chain ^34–36^. Zebrafish orthologs are highly homologous to human type IV collagen chains ^37^, and so it appears unlikely the zebrafish would synthesise a unique trimer to mammalian systems, however our inability to detect *col4a5* expression by *in situ* hybridisation means further analysis is required to determine if *col4a5* is expressed in the zebrafish pronephric glomerulus. To assay for the presence of type IV collagen protein in the glomerulus, we performed immunofluorescence with a pan-collagen IV antibody (which detects all six type IV collagen chains) on cryo-sectioned 4 dpf larvae (Figure 4C). Localisation of type IV collagen was evident in the glomerulus, as well as in surrounding epithelial tissues.

### Generation of an Alport syndrome model in the NL-D3 zebrafish reporter

**Figure 5:**
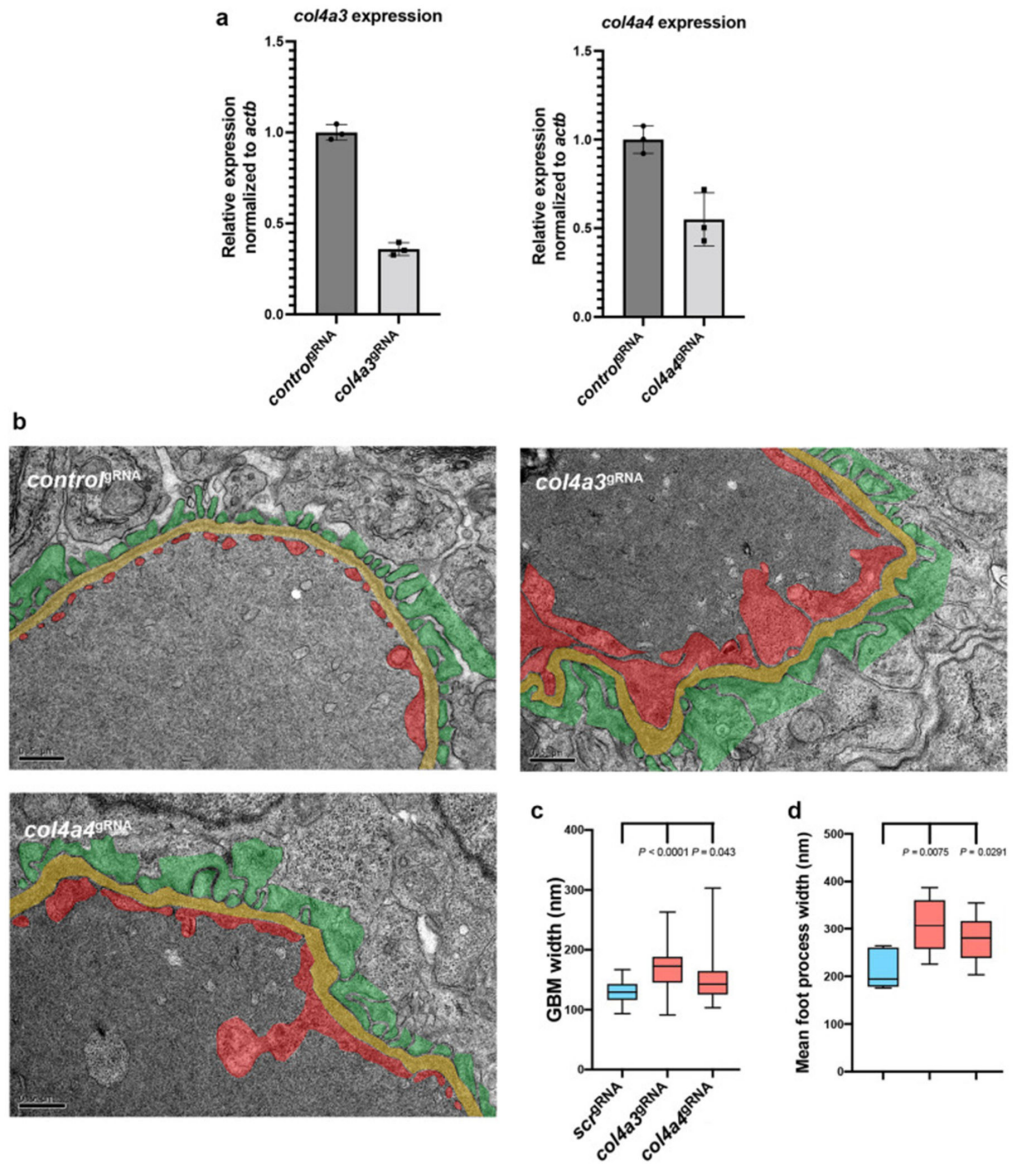
Depletion of *col4a3* or *col4a4* induces Alport syndrome phenotypes in zebrafish A) Bar chart showing RT-PCR analysis of *col4a3* and *col4a4* in wild-type versus respective crispant embryos (n=3). B) Panels show TEM images of the glomerular filtration barrier in control *scr*, *col4a3* and *col4a4* crispants (scale bar = 500 nm). Podocytes are pseudo-coloured green, GBM is yellow, and endothelium is red. C) Box and whisker plot showing the average GBM width in control *scr*, *col4a3*, and *col4a4* crispants. D) Box and whisker plot showing the average foot process width in control *scr*, *col4a3*, and *col4a4* crispants.

Given the distinct glomerular expression of *col4a3* and *col4a4* in the zebrafish, we proceeded to deplete these genes using a CRISPR-Cas9 method that yields knockout-like phenotypes in F0 embryos (termed crispants) ^12^. This approach robustly induced mutagenesis in *col4a3* and *col4a4* when analysed by TIDE (Figure S6) and individual allele analysis (Figure S7). However, the single allele analysis showed that, unlike the findings of Wu *et al* (2018), high rates of mutagenesis in all four gRNAs was not observed with this method. Only one gRNA caused reproducible indels in *col4a3* (with two out of the three indels being in the splicing site for exon 34 of col4a3), and two gRNAs caused indels in *col4a4*. Therefore it is possible that a number of our crispants represent heterozygous animals, and so are more representative of phenotypes observed in autosomal recessive Alport Syndrome ^38^. RT-PCR analysis demonstrated transcripts for *col4a3* and *col4a4* were reduced when each gene was targeted using gRNAs (n=3, Figure 5A). Ultrastructural analysis of the glomerular filtration barrier by transmission electron microscopy (TEM) identified significant defects in the glomerular capillary wall of the collagen IV crispants (Figure 5B – for non-annotated TEM views of controls and *col4a3/ col4a4* crispants see Figure S8). Quantification of average GBM width showed *col4a3* and *col4a4* crispants had thicker GBMs (Figure 5C) and average foot process length was increased, indicating effacement (Figure 5D). These glomerular ultrastructure changes phenocopy those observed in human patients with Alport syndrome and knockout mouse models of Alport syndrome ^39,40^.

**Figure 6:**
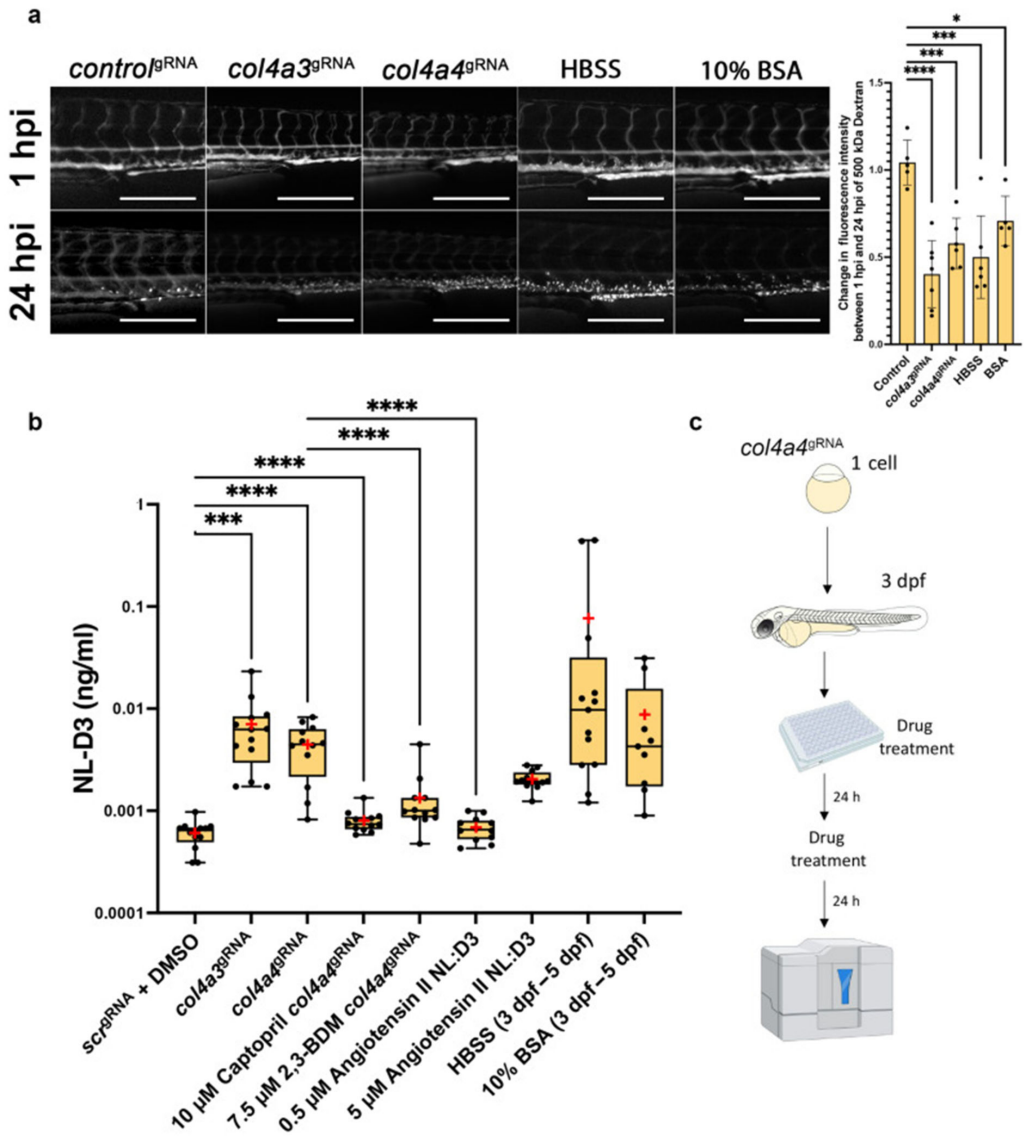
Alport zebrafish display proteinuria which can be alleviated by reducing intraglomerular force A) Panels showing 4 dpf embryos injected with a 500 kDa FITC-conjugated dextran at 1 hour post injection (hpi) and 24 hpi after the treatments shown (*control*^gRNA^ n=5, *col4a3*^gRNA^ n=6, *col4a4*^gRNA^ n=6, HBSS n=5, 10% BSA n=5). Scale bar = 250 μm. Bar chart to the right shows the change in fluorescence intensity measured in the dextran-injected embryos at 24 hpi compared to 1 hpi. B) Box and whisker plot showing amounts of NL-D3 produced into embryo medium in control *scr* (n=13), *col4a3* (n=13) and *col4a4* (n=14) crispants. The effect of the angiotensin converting enzyme inhibitor (ACEi) captopril (n=12) and 2,3-Butanedione monoxime (2,3-BDM, n=12) on proteinuria in *col4a4* crispants is also shown. Angiotensin II (n=12), HBSS (n=12), and 10% BSA (n=9) all increased proteinuria in *NL-D3* zebrafish embryos. Median is shown as a line and mean is shown as a red cross-hair. C) Schematic showing the experimental pipeline for assaying the effects of chemicals in zebrafish *NL-D3* embryos.

We next wished to determine if *col4a3*- and *col4a4*-deficient zebrafish model had increased levels of proteinuria, which is a feature of Alport syndrome. Depletion of *col4a3* (n=7) or *col4a4* (n=6) by CRISPR-Cas9 resulted in clearance of a 500 kDa FITC-conjugated dextran much faster than controls (n=5) (Figure 6A), demonstrating glomerular dysfunction in these crispants. We therefore determined if *col4a3* or *col4a4* crispants showed proteinuria in our transgenic *NL-D3* reporter line. This analysis showed that knockdown of either *col4a3* or *col4a4* in zebrafish crispants induced proteinuria (~12-fold for *col4a3* crispants n=13, ~7.4-fold for *col4a4* crispants n=12) when compared to *control*^gRNA^-injected embryos (n=13) (Figure 6B). In conclusion, knockdown of either *col4a3* or *col4a4* caused ultrastructural defects in the glomerular filtration barrier and induced proteinuria, recapitulating the phenotypes of pathogenic variants in Alport genes in both mouse knockout models and human patients.

### Pharmacological amelioration of proteinuria in zebrafish Alport crispants

Given the zebrafish *NL-D3* transgenic reporter line can be used for assaying glomerular dysfunction where proteinuria is a key clinical feature, such as in Alport syndrome, we next attempted to rescue glomerular filtration using chemical approaches to determine the potential for this system for compound screening. *col4a4* crispants were used as a model of Alport syndrome for these experiments. In *col4a4* crispants, captopril was used from 3 dpf to 5 dpf to reduce systemic blood pressure, and we assayed for proteinuria from 4 dpf to 5 dpf (Figure 6B). Captopril is an angiotensin-converting enzyme inhibitor (ACEi) and has previously been used in zebrafish to lower blood pressure ^41^, we hypothesised that reducing systemic blood pressure will also reduce intraglomerular pressure. We found that captopril treatment had no effect on un-injected zebrafish embryos (data not shown) but almost entirely prevented proteinuria in *col4a4* crispants (n=12, Figure 6B). We also used 2,3-butanedione 2-monoxime (2,3-BDM) to slow the heart rate in zebrafish embryos from 3 dpf to 5 dpf. We hypothesised that a lower heart rate would lead to reduced glomerular perfusion, thereby reducing glomerular filtration. In wild-type zebrafish embryos, a dose response analysis showed 2,3-BDM at a concentration of 7.5 μM was sufficient to significantly lower heart rate (Figure S9). Incubation of *col4a4* crispants with this concentration of 2,3-BDM lowered proteinuria in these animals to a level that was still ~2.2-fold higher than controls but ~3.3-fold lower than untreated *col4a4* crispants (n=12, Figure 6B).

Using the *NL-D3* reporter line, we also detected the effects of increasing systemic blood pressure on zebrafish embryos. Angiotensin II is a peptide hormone that increases blood pressure by inducing vasoconstriction. We found that 0.5 μM angiotensin II did not cause proteinuria in *NL-D3* embryos, however 5 μM angiotensin II resulted in a ~3.4-fold increase in proteinuria (n=12). Similarly, injection of Hank’s Balanced Salt Solution (HBSS) or 10% Bovine Serum Albumin (BSA) caused a dramatic increase in proteinuria in the *NL-D3* reporter embryos. HBSS is a saline solution that upon injection causes water influx into the blood vasculature and has been shown to expand glomerular capillaries ^42^. Thus, we hypothesised that the injection of HBSS creates an acute mechanical loading in the glomerulus that would cause proteinuria. In support of this we observed increased clearance of a 500 kDa FITC-conjugated dextran (n=6, Figure 6A). In HBSS-injected NL-D3 embryos, we detected a ~128-fold increase in luminescence (n=13, Figure 6B). Human albumin solution is given in the clinical setting to expand the circulation. Injection of a high concentration of BSA solution (10%) into zebrafish embryos was performed to increase glomerular perfusion. We again observed an increase in both clearance of a 500 kDa FITC-conjugated dextran (n=5, Figure 6A) and in NL-D3 proteinuria (n=9), which was ~15-fold higher than non-treated controls (Figure 6B).

Overall, these data confirm that the *NL-D3* reporter line can detect proteinuria associated with changes in systemic blood pressure and glomerular perfusion. They also highlight the potential of this new tool in kidney function therapeutic screening.

## Discussion

Here, we describe a new high-throughput tool for the quantification of proteinuria in the zebrafish model. We find that the *NL-D3* transgenic reporter can detect changes in both glomerular and proximal tubular function. Our investigations into the impact of chemical agents on wild-type and genetically modified kidneys show that significant changes in proteinuria can be detected. Given the simplicity of our *NL-D3* reporter, it is likely to have applications in future mutation and drug compound screening.

We have assessed the *NL-D3* reporter to ascertain any limitations with the line. Given the reporter is a transgenic system, animal-to-animal and generation-to-generation variability in expression of the transgene should be considered. To reduce the impact of transgene expression variation, we use three embryos per well instead of one embryo per well. After taking these measures the variability in wild-type embryos was negligible (deviation less than 10% from the mean). The brightness of the nanoluciferase assay system also means that if any embryos die or are accidentally destroyed during pipetting, the embryo medium from these wells should not be read. To reduce the impact of high readings, we also used opaque 96-well plates to minimise any bleed-through from luminescence in neighbouring wells. We also found that the NL-D3 reporter is more sensitive at later time-points. Severe tubular dysfunction caused by *lrp2a* depletion, led to detectable NL-D3 proteinuria between 48-72 hpf. However, glomerular dysfunction caused by depletion of *nphs1* did not cause statistically significant increases in NL-D3 proteinuria at this time-point. This is likely due to the glomerulus not being fully mature until 4 dpf, and so we suggest use of the NL-D3 reporter be restricted to later than 3-4 dpf for glomerular studies. Furthermore we did not observe NL-D3 proteinuria in *ocrl* morphants between 48-72 hpf, only later between 4-5 dpf. This observation highlights the need to perform preliminary experiments at a range of developmental time-points to ensure reliable data is acquired when using the NL-D3 proteinuria reporter system.

We expected the NL-D3 protein to readily pass through the glomerular filter and be required to be reabsorbed in the proximal tubule via megalin-mediated endocytosis. However, the glomerulus must also act as a partial barrier to NL-D3 given that we find genetic or chemical targeting of glomerular filtration induces an increase in the presence of NL-D3 in the embryo medium. Lower-than-expected filtration of NL-D3 mimics the glomerular barrier function to serum albumin, which was thought to be small enough to pass through the filter, but has a low sieving coefficient (0.00062) ^43^. Recent evidence suggests that the podocyte glycocalyx and the extent of GBM compression are important for the size selectivity of the glomerular filter ^2,3^. Thus, we speculate that these barriers prevent passage of NL-D3 via simple diffusion through the glomerular filter in the healthy state. However, when podocyte effacement occurs (as we see in our TEM images of Alport crispant zebrafish) the GBM compression and slit diaphragm are lost and NL-D3 is more readily able to pass through the filter, saturating the reabsorption pathways in the tubules, and leading to the proteinuria we detect.

In our studies we developed a new model of Alport syndrome that phenocopies many of the disease features found in human patients. The phenotypes in the glomerulus of Alport zebrafish were not as severe as observed in human patients, though this is likely due to the embryos being just 5 days old. Therefore, depletion of *col4a3* or *col4a4* in zebrafish is a model of the early stages of Alport syndrome. We have shown that *col4a3* and *col4a4* are expressed in the zebrafish glomerulus by *in situ* hybridisation. We were unable to detect *col4a5* in the glomerulus by *in situ* hybridisation, which may be due to the technical difficulties such as probe penetration. However, we cannot rule out that zebrafish are distinct in their production of col4a5. We consider this is unlikely as specific human and zebrafish chains cluster together when their sequences are analysed ^37^ (highlighting their conservation), and biochemical analyses of the human a3a4a5(IV) heterotrimer suggests a3(IV) and a4(IV) chains are only able to trimerise with one a5(IV) chain ^36^. Future studies will be able to more rigorously determine if *col4a5* is expressed in the pronephric glomerulus in zebrafish, and if it is not then an understanding of which type IV collagen chains form in the pronephric GBM at this larval stage may yield interesting insight into the evolution of type IV collagens and their roles in development. For this study, we used a combinatorial gRNA approach to introduce indels in F0 crispant embryos ^12^, which is not suited to establishing stable lines due to each gene being exposed to 4 gRNAs, making genotyping exhaustive and increasing the chance of off-target effects. However, the *col4a5 dragnet* mutant can survive to adulthood ^37^ and the restricted expression of *col4a3* and *col4a4* suggests a stable set of Alport zebrafish lines will be viable to adulthood, and these mutant lines could be used to confirm the phenotypes observed here in crispants. A stable line could also be used for a drug screen to discover chemicals that ameliorate proteinuria associated with Alport syndrome.

In summary, our data shows the development of a nanoluciferase based reporter for proteinuria in the zebrafish system. Given the advantages of this system in terms of experimental usability and the feasibility of high-throughput screens, we anticipate this new proteinuria reporter will be a valuable addition to the toolkit of kidney researchers.

## Supplementary Material

Supplementary File

Supplementary Figure

## Translational Statement

The zebrafish has become a useful tool for modelling kidney disease. However, proteinuria, an important clinical indicator of kidney dysfunction, is not easily detected in the zebrafish system. Here, we describe a novel transgenic zebrafish line that measures proteinuria associated with both glomerular and tubular dysfunction. This new resource will impact future treatments for patients by permitting rapid and economical pre-clinical screening of drugs to treat kidney disease associated with glomerular or tubular dysfunction.
