# Supplementary material for: A novel nanoluciferase transgenic reporter to measure proteinuria in zebrafish
Source: Kidney Int. Author manuscript; Available in PMC 2023 Mar 4. (PMC7614274; doi:10.1016/j.kint.2022.05.019)
Supplement: Supplementary File [file EMS170654-supplement-Supplementary_File.pdf]

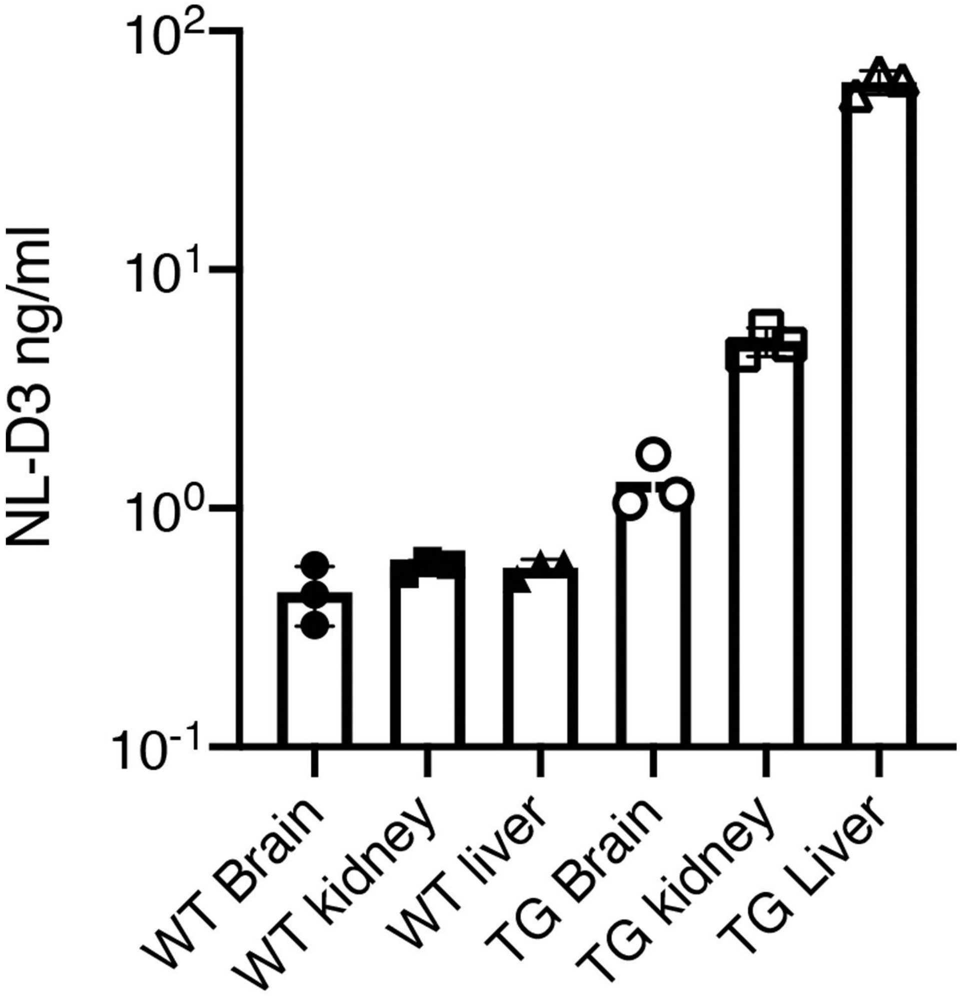

Figure S1: Comparison of luminescence in wild-type versus *NL-D3* adult tissue Histogram showing the relative luminescence intensities of brain, kidney and liver tissue extracted from wild-type (WT) and *NL-D3* (TG) adult zebrafish.

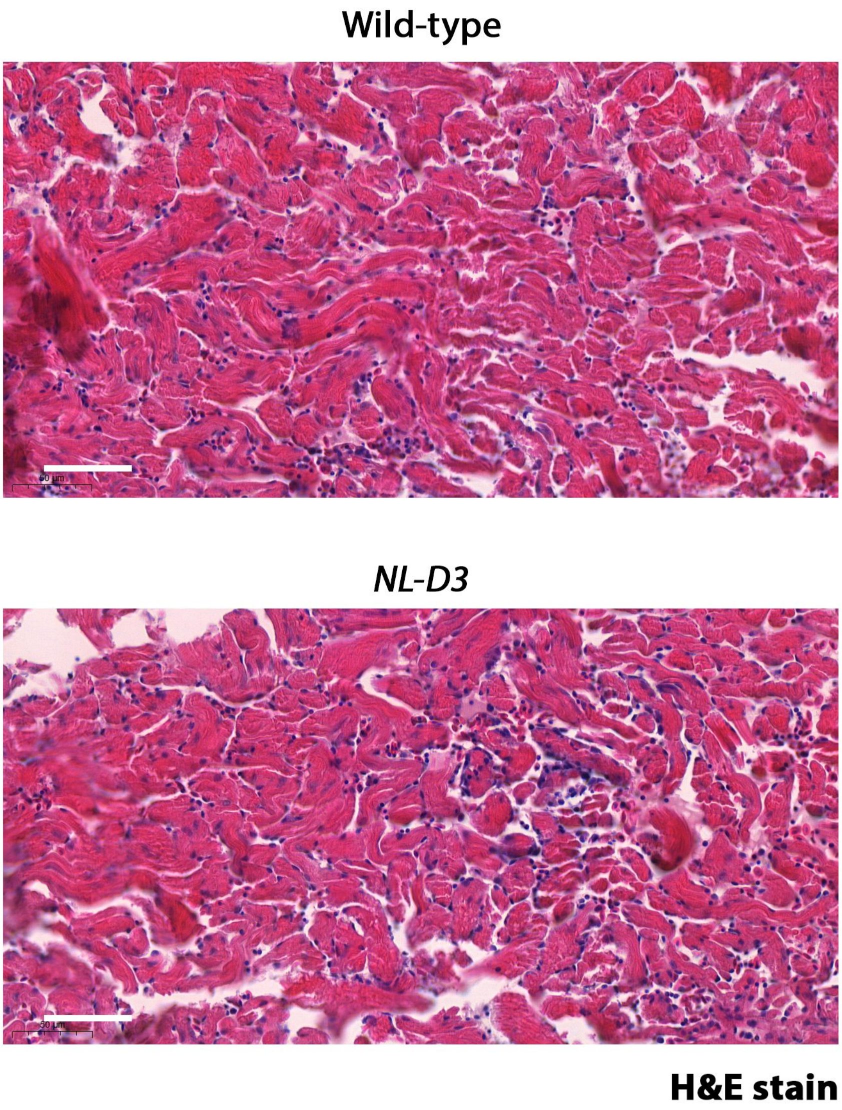

Figure S2: Histology of adult liver in wild-type and *NL-D3* transgenic zebrafish Panels show liver sections stained with H&E at ~3 months of age. Scale bar = 50 µm

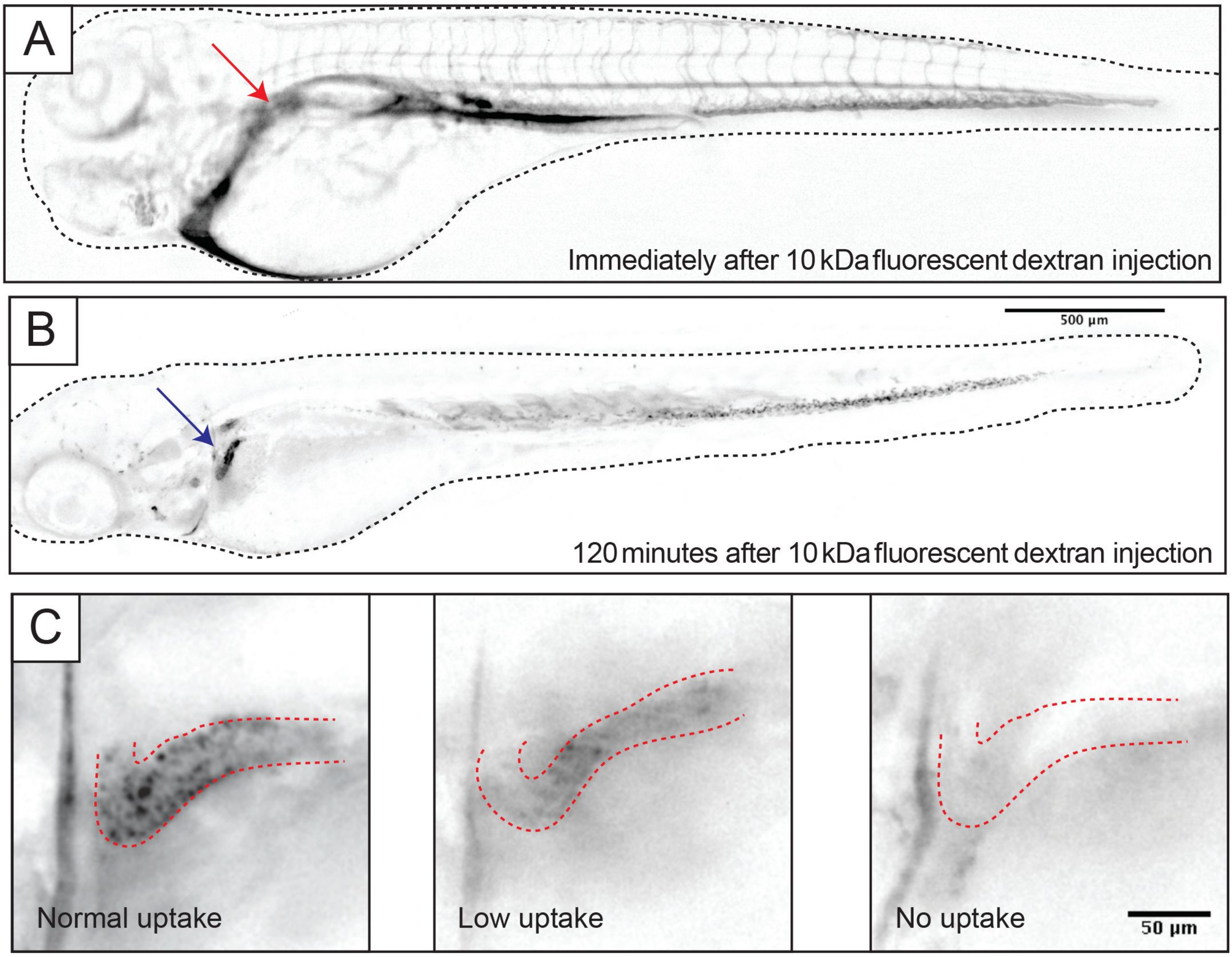

Figure S3: Dextran uptake classification A) Lateral view of a 4 dpf zebrafish embryo immediately after being injected with a 10 kDa fluorescent dextran into the common-cardinal vein. B) Lateral view of a 4 dpf zebrafish embryo 2 hours after being injected with a 10 kDa fluorescent dextran into the common-cardinal vein. C) Three panels showing lateral views of fluorescent dextran uptake in the proximal pronephric tubule. Panel on left shows normal uptake, middle panel shows low uptake, and right panel shows no uptake.

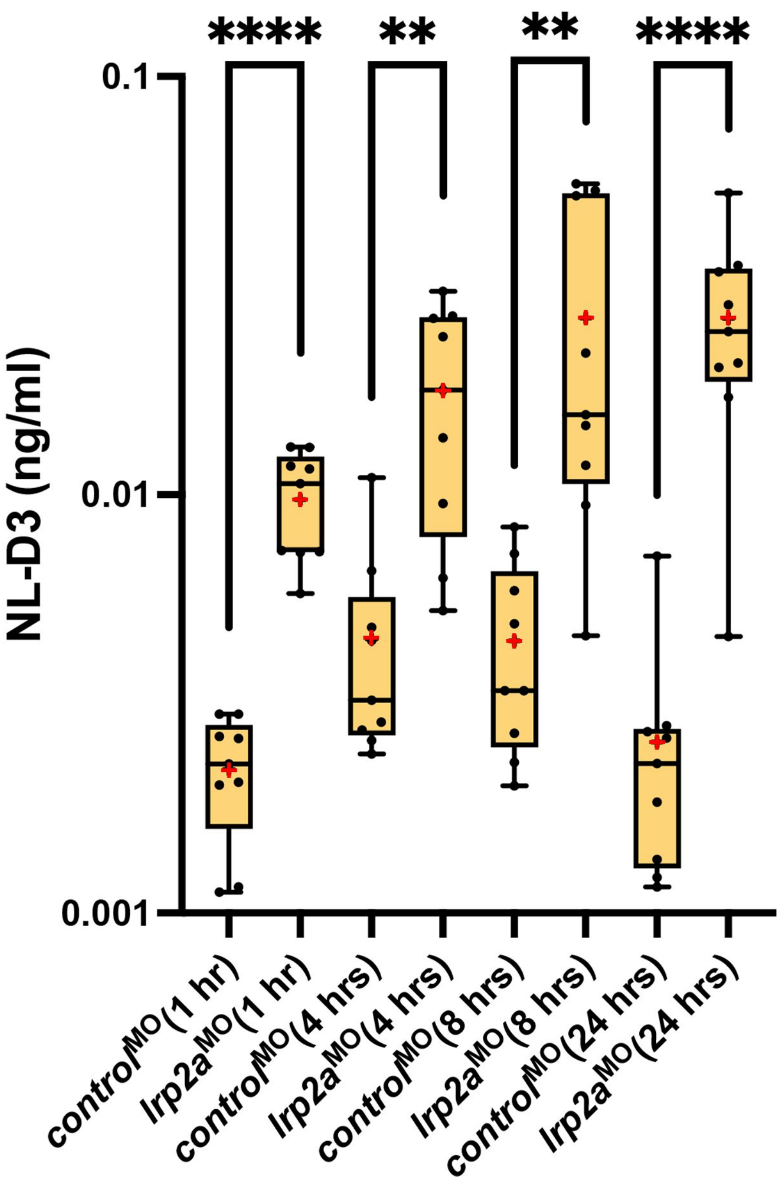

Figure S4: NL-D3 proteinuria can be observed after a short incubation time Box and whisker plot showing the amount of NL-D3 present in the embryo medium of control and *lrp2a* morphants after 1 hour, 4 hours, 8 hours and 24 hours. Median is shown as a line and mean is shown as a red cross-hair.

A

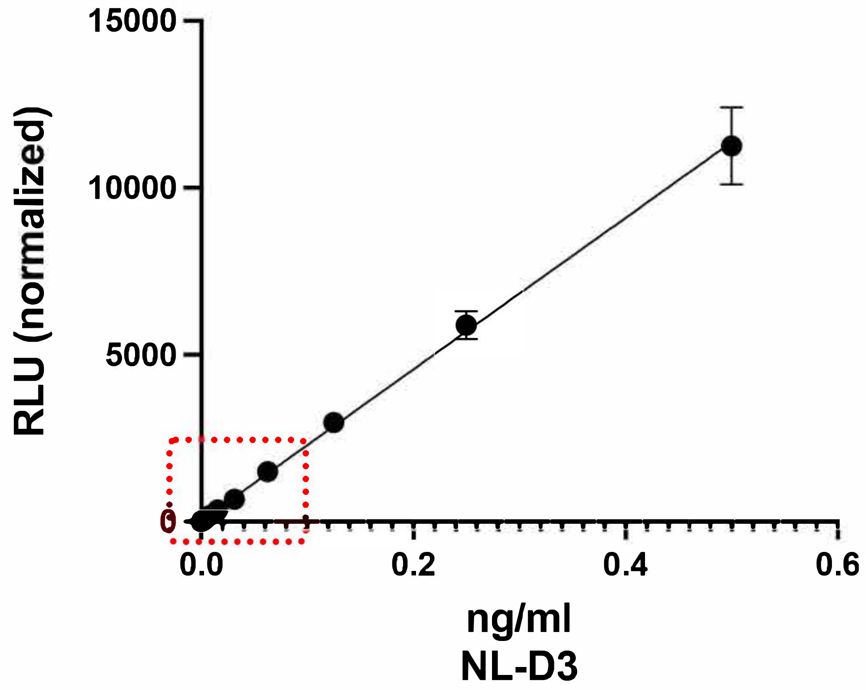

B

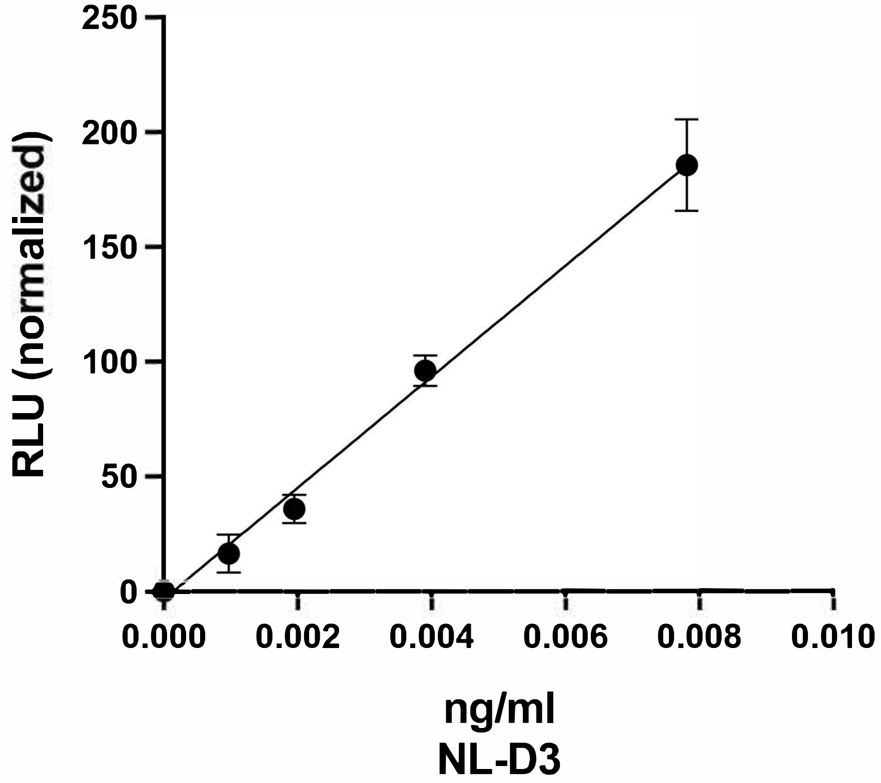

Figure S5: Standard curve data A) Graph showing standard curve curated from data collected analysing 0.5 ng/ml to 0.0005 ng/ml. B) Close up visualisation of the graph in (A) in the region of the red-dotted box to highlight the lower concentrations of NL-D3 where most data points were collected.

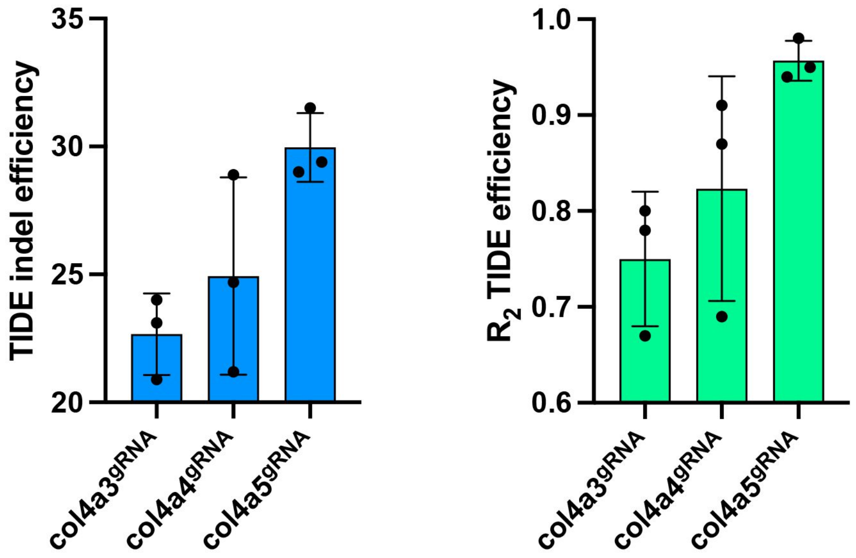

Figure S6: TIDE analysis shows mutagenesis at gRNA site 1 in the Alport zebrafish crispants Histogram on the left shows the TIDE indel efficiency (calculated with the algorithm described on https://tide.nki.nl/) for three biological replicates induced by gRNA 1 for the col IV crispant zebrafish embryos. Histogram on the right shows the R_2 values for the corresponding indel efficiencies.

col4a3

gRNA 1

```
ATGTGTAGGGTAAAGAAGGTCGGAGCATGGGAATACCTGGACCCAGCGGT
ATGTGTAGGGTAAAGAAGGTCGGAGCATGGGAATACCTGGACCCAGCGGT
ATGTGTAGGGTAAAGAAGGTCGGAGCATGGGAATACCTGGACCCAGCGGT
ATGTGTAGGGTAAAGAAGGTCGGAGCATGGGAATACCTGGACCCAGCGGT
ATGTGTAGGGTAAAGAAGGTCGGAGCATGGGAATACCTGGACCCAGCGGT
ATGTGTAGGGTAAAGAAGGTCGGAGCATGGGAATACCTGGACCCAGCGGT
```

gRNA 2

```
CATCACAGGACCCAAAGGTCGTGATGGCCAACGAGGGGAACAAGGACCCC
CATCACAGGACCCAAAGGTCGTGATGGCCAACGAGGGGAACAAGGACCCC
CATCACAGGACCCAAAGGTCGTGATGGCCAACGAGGGGAACAAGGACCCC
CATCACAGGACCCAAAGGTCGTGATGGCCAACGAGGGGAACAAGGACCCC
CATCACAGGACCCAAAGGTCGTGATGGCCAACGAGGGGAACAAGGACCCC
CATCACAGGACCCAAAGGTCGTGATGGCCAACGAGGGGAACAAGGACCCC
```

gRNA 3

```
TATCTCAGGGTGAAATAGCATTTGGCTGCCCAGGGGCAAGGGGTAGAGAA
TATCTCAGGGTGAAATAGCATTTGGCTGCCCAGGGGCAAGGGGTAGAGAA
TATCTCAGGGTGAAATAGCATTTGGCTGCCCAGGGGCAAGGGGTAGAGAA
TATCTCAGGGTGAAATAGCATTTGGCTGCCCAGGGGCAAGGGGTAGAGAA
TATCTCAGGGTGAAATAGCATTTGGCTGCCCAGGGGCAAGGGGTAGAGAA
TATCTCAGGGTGAAATAGCATTTGGCTGCCCAGGGGCAAGGGGTAGAGAA
```

gRNA 4

```
AGAACTTGGTCCTCCTGGCCTACCAGGTGACTCATTCAACAGTTACATTT
AGAACTTGGTCCTCCTGGCCTACCAGGTTAATCATTCAACAGTTACATTT
AGAACTTGGTCCTCCTGGCCTAC--GGTGACTCATTCAACAGTTACATTT
AGAACTTGGTCCTCCTGGCCTAC--GGTGACTCATTCAACAGTTACATTT
AGAACTTGGTCCTCCTGGCCTACCAGGTTAATCATTCAACAGTTACATTT
AGAACTTGGTCCTCCTGGCCTAC--GGTGACTCATTCAACAGTTACATTT
```

col4a4

gRNA 1

```
GGCTGAGATGACACGCCCCCACCGAATCGCCTCGACCAACCTCTTTTTCT
GGCTGAGATGACACGCCCCCACC--------------AACCTCTTTTTCT
GGCTGAGATGACACGCCCCCACC--------------AACCTCTTTTTCT
GGCTGAGATGACACGCCCCCACCG-----CCTCGACCAACCTCTTTTTCT
GGCTGAGATGACACGCCCCCACCGAATCGCCTCGACCAACCTCTTTTTCT
GGCTGAGATGACACGCCCCCACC--ATCGCCTCGACCAACCTCTTTTTCT
```

gRNA 2

```
GGTATACCTGGACCTTCTGGTGATCCAGGACTATCTTTACCAGGTTTGAG
GGTATACCTGGACCTTCTGGTGATCCAGGACTATCTTTACCAGGTTTGAG
GGTATACCTGGACCTTCTGGTGATCCAGGACTATCTTTACCAGGTTTGAG
GGTATACCTGGACCTTCTGGTGATCCAGGACTATCTTTACCAGGTTTGAG
GGTATACCTGGACCTTCTGGTGATCCAGGACTATCTTTACCAGGTTTGAG
GGTATACCTGGACCTTCTGGTGATCCAGGACTATCTTTACCAGGTTTGAG
```

gRNA 3

```
CCAGGTCCACCGGGAACACCGGGGTATCCGGGTCTGAATGGACCTCCAGG
CCAGGTCCACCGGGAACACCGGGGTATCCGGGTCTGAATGGACCTCCAGG
CCAGGTCCACCGGGAACACCGGGGTATCCGGGTCTGAATGGACCTCCAGG
CCAGGTCCACCGGGAACACCGGGGTATCCGGGTCTGAATGGACCTCCAGG
CCAGGTCCACCGGGAACACCGGGGTATCCGGGTCTGAATGGACCTCCAGG
CCAGGTCCACCGGGAACACCGGGGTATCCGGGTCTGAATGGACCTCCAGG
```

gRNA 4

```
GGCGACAGTGGTGAATCTATTTGTACGCCAGGGCGAACAGGTGAGAGAGG
GGCGACAGTGGTGAAT----------GCCAGGGCGAACAGGTGAGAGAGG
GGCGACAGTGGTGAAT----------GCCAGGGCGAACAGGTGAGAGAGG
GGCGACAGTGGTGAAT----------GCCAGGGCGAACAGGTGAGAGAGG
GGCGACAGTGGTGAATCTATTTGTA-GCCAGGGCGAACAGGTGAGAGAGG
GGCGACAGTGGTGAATCTATTTGTA-GCCAGGGCGAACAGGTGAGAGAGG
```

Figure S7: Single allele analysis of *col4a3* and *col4a4* crispants Sequences show wild type on top row with five sequencing analyses repeats of crispants below. *col4a3* sequences around the 4 gRNA sites are shown on the left, *col4a4* sequences around the 4 gRNA sites are shown on the right. Indels in individual alleles taken from mosaic animals were not detected in all four gRNAs. Only gRNA 4 in *col4a3* identified mutagenic alleles with two indels identified in all five clones analysed. In *col4a4*, gRNA1 and gRNA4 created indels.

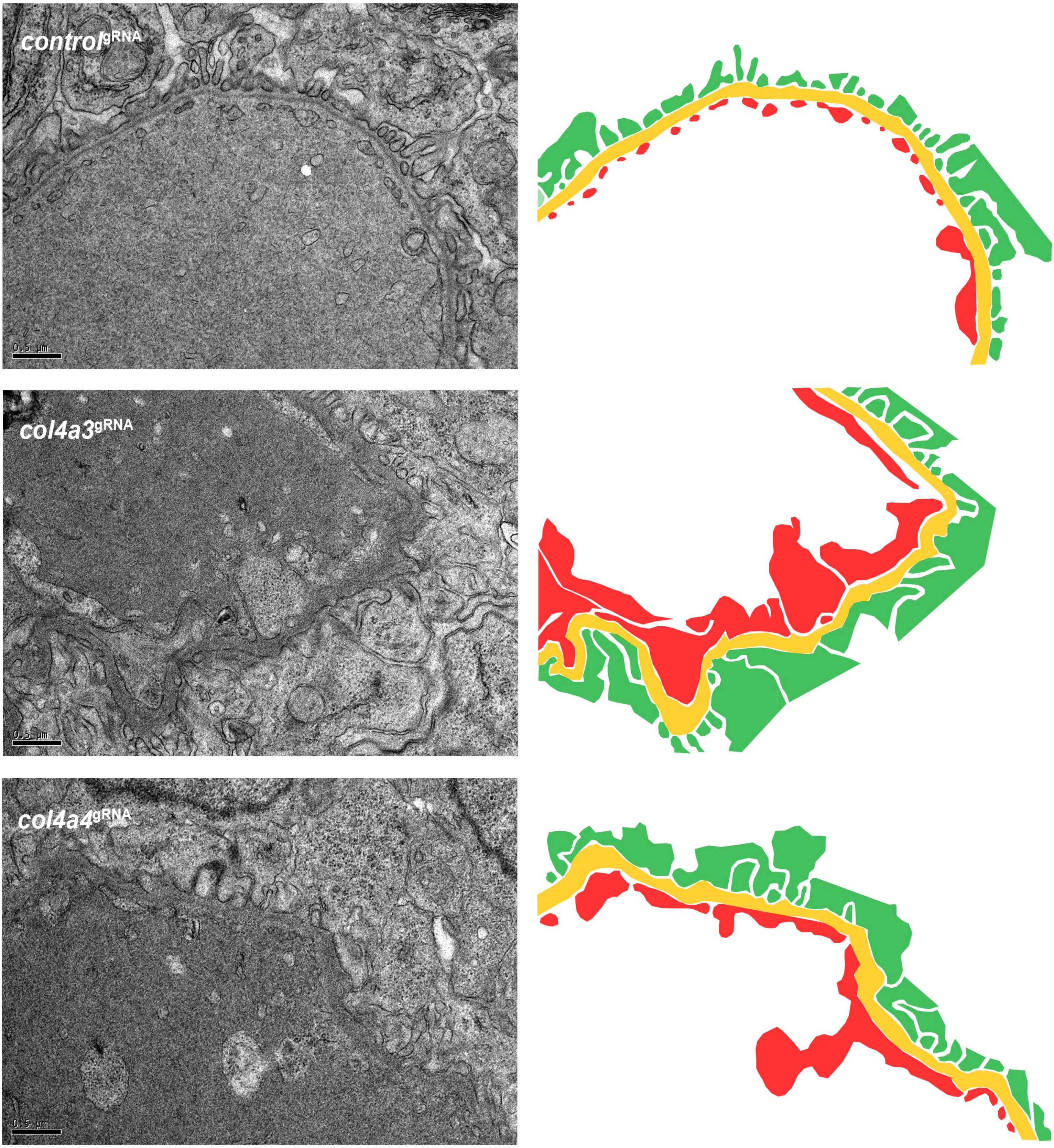

Figure S8: Non-annotated TEM images of glomeruli in control and *col4a3* and *col4a4* crispants Panels show cross-section of glomerular filtration barrier under TEM. The pseudo-colouring from Figure 5 is shown as a separate image on the right. Podocyte foot processes (green), GBM (yellow), and endothelium (red) are highlighted. Scale bar = 500 nm

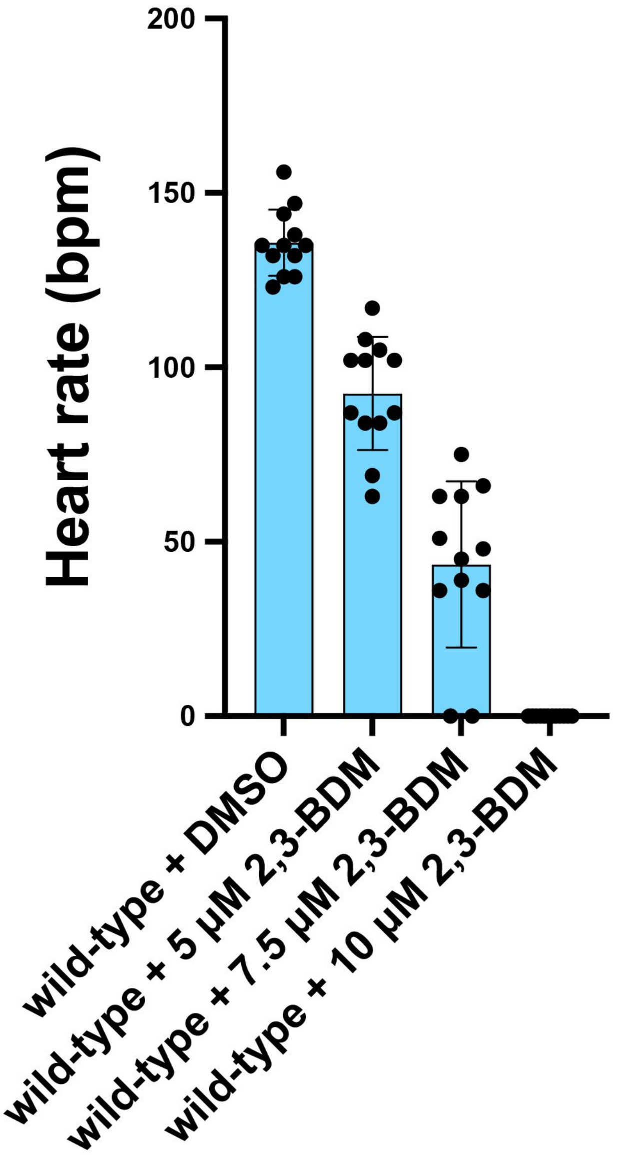

Figure S9: Increasing 2,3-BDM dose results in increasing effect on heart rate in zebrafish embryos Histogram shows heart rate (bpm) of wild-type embryos treated for 1 hour with vehicle (DMSO) or 2,3-BDM of varying concentrations highlighted.

Extended Supplementary Methods

Zebrafish husbandry

Zebrafish were maintained and staged according to established protocols [13] and in accordance with the project licenses of Martin Lowe (70/9091) and Rachel Lennon (P1AE9A736) under the current guidelines of the UK Animals Act 1986. Embryos were collected from group-wise matings of wild-type AB Notts, or *γ-crystallin:mcherry/fabp10a:NL-D3* fish.

Molecular Cloning

The cDNA for rat RAP was a gift from Prof. Thomas Willnow (Max Delbruck Centre for Molecular Medicine, Berlin). The nanoluciferase (NL) cDNA was from Promega, with an N-terminal fusion of the IL-6 signal sequence [14]. The D3 fragment of RAP (amino acid numbers 206-319, lacking the carboxy-terminal HNEL ER retention sequence) was cloned downstream of the NL cDNA prior to the stop codon to create a C-terminal fusion. NL-D3 was cloned by PCR into the pTrcHis bacterial expression vector for preparation of recombinant His-tagged protein in *E. coli*. For generation of zebrafish transgenics, the Gateway cloning method was used [15]. NL-D3 cDNA was cloned into the Gateway p3 vector. The p1 vector contained the γ-Crystallin promoter driving mCherry expression [16] and p2 vector contained the liver specific *fabp10a* promoter [17]. The p1-p3 plasmids were combined with the p4-I-SceI SAR-CH4 Tol2 destination vector [18] to make the final transgenesis construct.

Zebrafish transgenesis

5 µl of 80 ng/µl DNA of the transgenesis construct were mixed with 5 µl of 50 ng/µl *tol2* transposase mRNA and 1 µl of 10X phenol red. All solutions were prepared fresh immediately before injection. Each single cell stage embryo was oriented to allow access into the cell and 1 nl of this solution was injected directly into the cell using a microinjector. Successful transgenesis was confirmed in F0 embryos by selecting for mCherry expression in the lens at day 5, and founder fish were then grown to adulthood.

Preparation of recombinant NL-D3

His-tagged NL-D3 was expressed in *E. coli* BL21(De3) Codon Plus cells by induction with 1 mM IPTG for 16 hours at 18˚C. The protein was purified using Ni-NTA agarose using standard methods, snap frozen in liquid nitrogen, and stored at -80˚C until use.

Cell culture

Parental HEK293 EBNA cells or HEK293 EBNA cells stably expressing megalin mini-receptor (MmR4) were kindly provided by Prof. Thomas Willnow (Max Delbruck Centre for Molecular Medicine, Berlin). The cells were maintained at 37˚C in 5% CO_2 in DMEM containing 10% fetal bovine serum, penicillin (100 U/ml), streptomycin (0.1 mg/ml), and 0.35 mg/ml G418 without (parental) or with (MmR4 cells) an additional 1 µg/ml puromycin. For NL-D3 uptake, cells were washed with PBS, then serum-free DMEM before pre-incubating in serum-free DMEM for 30 min at 37˚C. Cells were then incubated in serum-free DMEM containing NL-D3 at 1 µg/ml for 30 min at 37˚C, washed 3x in PBS, and then lysed in Nano-Glo luciferase buffer (Promega) for assessment of luciferase activity.

Drug treatments

Drug treatments were performed on zebrafish embryos either by injection into the common cardinal vein (HBSS (Thermofisher #14170070, neat), BSA (ThermoFisher #B14, 10%)) or by incubation in the E3 embryo medium (captopril (Bio-Techne #4455/50, 10 µM), angiotensin (Eurogentec #AS20634, 0.5-5 µM)). For injection, embryos were grown to desired stage (3 dpf) and then anaesthetised in buffered tricaine methanesulfonate (MS-222) at 164 mg/L. Embryos were then placed in an injection chamber containing MS-222 and injected into the common cardinal vein with a microinjection needle. Embryos were then transferred to fresh E3 embryo medium to recover and grown to desired stage. For incubations, embryos were transferred with a transfer pipette to fresh E3 embryo medium containing the drug at the desired concentration. Control embryos were treated with the equivalent concentration of vehicle substance (DMSO).

For gentamicin and cisplatin treatments, previous methods described by Chen et al, (2020) were used. In brief, gentamicin (Sigma, #G1264) was re-suspended in a 0.9% NaCl solution to a concentration of 10 ng/µl. This was diluted to 6 ng/µl for injection, which was performed at 48 hpf into the common cardinal vein (either 0.5 nl or 1 nl injection volume). For cisplatin treatments, the drug was re-suspended directly into E3 embryo medium containing 0.01% methylene blue. Concentrations of 0.5 mM and 1.5 mM were exposed to embryos at 48 hpf for a period of four hours. Embryos were then washed three times in E3 medium and left to grow to the desired stage for proteinuria analysis.

Morpholino oligonucleotide treatments

For *lrp2a* and *ocrl* morpholino experiments, both morpholinos were diluted to a final concentration of 0.24 mM. For control experiments, 0.24 mM of a standard control morpholino oligonucleotide was used. For *lrp2a* and *ocrl* knockdowns, a *p53* morpholino was also co-injected at the same concentration (0.24 mM). For *nphs1* and *nphs2* morpholino experiments, final concentrations of 0.15 mM (*nphs1*^{MOex25}) and 0.25 mM (*nphs2*^{MOex3}) were used. Injection droplet sizes were 5 nl for all morpholino experiments, and if defrosting a morpholino, the oligonucleotide was heated at 65ºC for 10 minutes prior to adding to the injection mixture. All microinjections were into the cell compartment of one-cell stage embryos. Morpholino sequences are shown below;

standard control – 5' CCTCTTACCTCAGTTACAATTTATA 3'

p53 – 5' GCGCCATTGCTTTGCAAGAATTG 3'

lrp2a – 5' AATCAGTGCTTGTGGTTTACCTGGG 3'

ocrl – 5' AATCCCAAATGAAGGTTCCATCATG 3'

nphs1 – 5' TGCACCAACACGACTCACCTCTGCTC 3'

nphs2 – 5' TGTAGTCACTTTTGCAGACCTGGGCT 3'

CRISPR-Cas9 knockdowns

To genetically knockdown expression of gene products, we adopted the CRISPR-Cas9 approach described by [19]. gRNAs were order from Merck and resuspended in nuclease-free water to a concentration of 20 μM. For the injection mix, 4 μM of each gRNA was combined with Cas9 (NEB #M0646) and Cas9 buffer. This injection mixture was then injected into the cytoplasm of 1-cell stage NL-D3 transgenic embryos. Below are the gRNAs used in this work;

col4a3 gRNA1 - GGTCGGAGCATGGGAATACC

col4a3 gRNA1 - CGTTGGCCATCACGACCTTT

col4a3 gRNA1 - GAAATAGCATTTGGCTGCCC

col4a3 gRNA1 - TGTTGAATGAGTCACCTGGT

col4a4 gRNA1 - GATCCAGGACTATCTTTACC

col4a4 gRNA2 - CTTGAAGCCCTTTAGGGCCA

col4a4 gRNA3 - GGATACCCCGGTGTTCCCGG

col4a4 gRNA4 - TGGATCCCCCATTAACCCCA

control gRNA1 - CAGGCAAAGAATCCCTGCC

control gRNA2 - TACAGTGGACCTCGGTGTC

control gRNA3 - CTTCATACAATAGACGATG

control gRNA4 - TCGTTTTGCAGTAGGATCG

Fluorescent re-uptake assays

β-Lactoglobulin from bovine milk (Sigma, L3908) was resuspended in 1X PBS and labelled with Cy3 mono-reactive dye (Amersham, PA23001) according to the manufacturer's instructions. 5 dpf embryos were anesthetised in E3 and MS222 (0.4 mg/ml) solution and then transferred to a 1% agarose injection dish. Larvae were injected in the common cardinal vein

with 1 nl of Cy3-β-Lactoglobulin (5 mg/ml) and/or Alexa fluor™ 488 10 kDa dextran (D22910, Thermo Fisher, at 2 mg/ml) diluted in sterile H_2O and successful injections were screened for on a Leica Mz10F fluorescent stereomicroscope. Uptake in the proximal tubule was visualised <1 hour post injection.

For glomerular function experiments, 1 nl of 5 mg/ml 500 kDa FITC-conjugated Dextran (ThermoFisher, D7136) was injected into the common cardinal vein at 4 dpf. Images of the posterior trunk intersegmental vessels roughly at the position of the cloaca were taken one hour post injection (hpi) on a Leica Mz10F fluorescent stereomicroscope. 24 hours later the embryos were re-imaged. All images were taken at the same magnification, laser power, and exposure time.

Standard curve generation

Fifty microlitres of serially diluted recombinant NL-D3 protein in PBS (0.5 ng/ml to 0.0005 ng/ml) were placed in the wells of a 96-well plate. The Nano-Glo® luciferase assay system (Promega #N1110) was used, with 50 µl of this solution added to each well containing recombinant NL-D3, or the buffer only blank. The plate was then placed in a Flexstation® 3 Multi-mode microplate reader and luminescence was measured by reading each well for 1 second. Data was collected in relative luminescence units (RLU) using SoftMax® Pro software. Using RLU values of each serial dilution minus the blank, a standard curve of known concentrations of recombinant NL-D3 relative to RLU was established. Conversion of RLUs to NL-D3 amount is not fundamental to infer changes in proteinuria in the reporter, but the empirical standard curve data can be provided upon request, with which users can interpolate their RLU readings in GraphPad Prism and generate NL-D3 ng/ml readings.

Zebrafish proteinuria reporter assay

The pipeline of the assay for proteinuria in *NL-D3* zebrafish is shown in the schematics accompanying Figures 2B and 6B. Embryos were grown to 4 dpf, then three embryos per well were placed in one well of a 96-well dish (Figure 2B). E3 embryo medium was removed and replaced with 200 µl fresh E3. 24 hours later 50 µl of E3 medium was removed from each well and placed in the corresponding well of a fresh opaque 96-well plate. 50 µl of substrate from the NanoGlo® Luciferase Assay System (Promega #N1110) was then added to each well. Plates were then briefly spun down at 700 rpm for 1 minute and then immediately assayed for luminescence on a Flexstation 3 multi-mode microplate reader. Softmax Pro 5.4 software (Molecular Devices) was used to detect luminescence. Endpoint analysis was selected with a 1000 millisecond integration of luminescence (RLUs). Opaque 96-well option was selected in 'Assay Plate', as well as Costar 96 opaque 3mL in 'Compound Source'.

RT-PCR

For RT-PCR, cDNA was diluted to 1 ng/µl and mixed with 7.8 µM of primers and added in equal volume to 2X *Power* SYBR^{TM} Green PCR Master Mix (Thermofisher #4367659). The primers used are described below;

actb F - TCACCACCACAGCCGAAAG

actb R - AGAGGCAGCGGTTCCCAT

col4a3 F - AACTTGTCGCTACGCCTCTC

col4a3 R - ATTGCCTCGCATACGGAACA

col4a4 F - CTGGCTTTAAGGGACCTCCG

col4a4 R - AAGCAGACTGTAGCCGTTCC

The PCR reaction was run on a Bio-Rad CFX96 Touch Real-Time PCR machine. All analyses of the data used the ∆∆Ct method of quantification. RT-PCRs were run in triplicate with -RT and -cDNA controls.

CRISPR mutation analysis

Embryos injected with gRNAs were grown to 24 hpf, dechorionated, then ten randomly selected embryos were placed in a 1.5 ml capped tube. All excess E3 embryo medium was removed and replaced with 50 μl of 50 μM NaOH. Embryos were then placed at 95˚C in a heating block for 15 minutes. After a brief spin, 5 μl of Tris-HCl pH 8 was added to neutralize the samples. The genomic DNA preps were then homogenized by pipetting before 1 μl was used in a PCR to amplify the region around the gRNA cut sight using the primers shown in the primer list below. For TIDE analysis, the PCR product was purified, ran on a gel to check size, and then diluted to 5 ng/μl before being sequenced. Wild-type *control*^{gRNA}-injected control ab1 files were used to compare crispants according to the TIDE guidelines [20]. TIDE analysis was performed online at https://tide.nki.nl/. For single allele analysis, PCR products were cloned using the TOPO Blunt kit (ThermoFisher, K280002) and transformed into BL21-Gold (DE3) competent cells (Agilent Technologies, 230132). Plasmid clones were isolated from bacteria using Qiagen Miniprep kit (27106X4) and sequenced by the Sanger method.

col4a3 TIDE F – tgccaggtgttcagggaaat

col4a3 TIDE R – aggctcctttggtcctgatg

col4a4 TIDE F – tttctcaccccactcctcac

col4a4 TIDE R – gatggcggtacacatggatg

col4a3 TOPO gRNA1 F – tgccaggtgttcagggaaat

col4a3 TOPO gRNA1 R – catcaggaccaaaggagcct

col4a3 TOPO gRNA2 F – ttttccacacgcacaaacaa

col4a3 TOPO gRNA2 R – cctcagggcctattagacct

col4a3 TOPO gRNA3 F – ggtgataagggtgtgaaaggg

col4a3 TOPO gRNA3 R – ccaccaaccaaattctccagg

col4a3 TOPO gRNA4 F – ggtcccaggtgatgaaggaa

col4a3 TOPO gRNA4 R – ggaagcccctttatgccagg

col4a4 TOPO gRNA1 F – tagctgaccacaccgttcat

col4a4 TOPO gRNA1 R – gatggcggtacacatggatg

col4a4 TOPO gRNA2 F – tgacagagggttgatgggtata

col4a4 TOPO gRNA2 R – accgtagctcaagagtgacc

col4a4 TOPO gRNA3 F – gtgaaaggagaaataggaccacc

col4a4 TOPO gRNA3 R – aattccctttggcccatgtg

col4a4 TOPO gRNA4 F – gttggaggatgaaactggtcc

col4a4 TOPO gRNA4 R – ccattgtctcctctctcccc

In situ hybridisation

Whole-mount in situ hybridisation on zebrafish embryos was performed as previously described [21]. Digoxigenin-labelled anti-sense riboprobes were made using T3 RNA polymerase transcription kits (Roche Diagnostics). Probe templates for zebrafish *col4a3, col4a4* and *col4a5* were generated by PCR amplification from cDNA taken from 4 dpf zebrafish embryos. The primers used were as follows:

F *col4a3* 5′-AAAGGGGCTTGTGATTGCAG-3′,

R *col4a3* 5′-GGATCCAATTAACCCTCACTAAAGGGATCTCCAGCTCTGCCTTGTT-3′;

F *col4a4* 5′-CAGAGGCTTAACAGGTCCCA-3′,

R *col4a4* 5′-GGATCCAATTAACCCTCACTAAAGGGTCCACAATCCCCAGGTTCTC-3′.

F *col4a5* 5’-GCCTATTGTCTTGAAGGGCG-3’

R *col4a5* 5’-GGATCCAATTAACCCTCACTAAAGGGACGGGAAGCGAAGTTACAGA-3’

A T3 anchor sequence (GGATCCAATTAACCCTCACTAAAGGG) on the 5’ end of the reverse primer was used to enable T3-mediated RNA synthesis from the purified PCR product. Following colorimetric assay, the embryos were treated with 100% Methanol for 10 minutes

with rocking to clear background labelling and were then fixed in 4% PFA for downstream imaging. Whole embryos were imaged on a Leica M205 FA upright stereofluorescence microscope, and transverse sections were imaged on an Olympus BX63 snapshot slide scanner microscope.

Cryosectioning and immunofluorescence

4 dpf zebrafish embryos were fixed in Dent's fixative (80% Methanol, 20% DMSO) or 4% PFA (for podocin staining) overnight at 4˚C. These embryos were then washed three times in 1X PBS before being placed in cryomolds. All excess PBS was removed with a pipettor and finally with a Kimwipe. The cryomold chamber was then filled with OCT compound. The embryos were then oriented in one direction in the OCT. A beaker of isopentane prechilled (for at least 30 minutes) in a polystyrene box filled with dry ice was prepared. The cryomold was carefully submerged into the isopentane to flash freeze the sample. Samples were then stored at -80˚C. Cryosectioning was performed in a Leica CM1950 cryostat, with 10 μm sections collected on coated glass slides. These collected sections were left to dry overnight at 4˚C and then were processed for immunostaining. Slides were washed four times in PBS containing 1% Triton X100 (PBSTr). They were then washed once with distilled water before being permeabilized in acetone for 8 minutes at -20˚C. Permeabilized sections were then washed in distilled water and four more times with PBSTr. The samples were then placed in blocking solution (5% BSA, 3% donkey serum in PBSTr) for at least one hour. The blocking solution was removed and replaced with the primary antibody in blocking solution. The primary antibodies used were NPHS2 (Abcam #ab50339; 1:250) and pan collagen IV (Abcam #ab6586; 1:250). Importantly, embryos fixed in PFA were used for the podocin staining and embryos fixed in Dent's fixative were used for the pan collagen IV staining. The primary antibodies were left on overnight at 4˚C and then washed at least 6x 1hr in PBSTr before being placed in secondary antibody Alexa Fluor 488 (1:500 in PBSTr) and left overnight again at 4˚C. The slides were then washed five times in PBS containing 0.05% Tween before being mounted with a coverslip using Prolong Diamond antifade mountant (ThermoFisher #P10144). Images were collected on a

Leica TCS SP8 AOBS inverted confocal using a *60X Plan Fluotar* objective. The confocal settings were as follows, pinhole *1 airy unit*, scan speed *1000Hz unidirectional*, format *1024 x 1024.* Images were collected *using the white light laser with 488nm (10%) laser lines.*

Wax sectioning and staining

For liver histological analysis, 3-month-old adult zebrafish were humanely killed by schedule 1 method and livers dissected. Isolated tissue was placed in 4% PFA overnight at 4˚C. Tissue infiltration was performed in a tissue processor and then embedded and mounted with paraffin wax. 5 μm sections were cut on a Leica RM2255 microtome. Sections were adhered to glass slides and dewaxed and stained with hematoxylin and eosin. Stained slides were coverslipped and imaged on a 3D Histec Pannoramic250 slide scanner.

Transmission electron microscopy

Samples were prepared according to protocols described previously [22]. Images were taken on T12 Biotwin transmission electron microscope. Distances were measured in Fiji/ImageJ, measurements for foot process width were taken and normalized to the length of the glomerular basement membrane. The total number of GBM width measurements for each sample was: *control* crispant, n=36; *col4a3* crispant, n=32; *col4a4* crispant, n=37; *col4a5* crispant, n=30. For foot process/ GBM length measurements, five TEM images per treatment were used and the total GBM length (nm) was measured. The number of foot processes along this GBM length was counted, then this number was divided by the GBM length. The mean ± SEM was calculated, and a student's t-test was performed using GraphPad Prism version 8.4.3 for 120 Windows, GraphPad Software, San Diego, California 121 USA, www.graphpad.com

Statistical analysis

The mean ±SD was calculated using GraphPad Prism version 9 for Windows, GraphPad Software, San Diego, California USA, www.graphpad.com. Statistical significance scores

were measured in GraphPad Prism version 9 using unpaired parametric Student's t test. P values in 95% confidence limits were characterised as significant, R squared scores from this statistical analysis was also noted to determine the size of the difference between the two compared datasets.
