# Supplementary material for: A novel nanoluciferase transgenic reporter to measure proteinuria in zebrafish
Source: Kidney Int. Author manuscript; Available in PMC 2023 Mar 4. (PMC7614274; doi:10.1016/j.kint.2022.05.019)
Supplement: Supplementary Figure [file EMS170654-supplement-Supplementary_Figure.pdf]

Figure S1: Comparison of luminescence in wild-type versus *NL-D3* adult tissue Histogram showing the relative luminescence intensities of brain, kidney and liver tissue extracted from wild-type (WT) and *NL-D3* (TG) adult zebrafish.

Figure S2: Histology of adult liver in wild-type and *NL-D3* transgenic zebrafish Panels show liver sections stained with H&E at ~3 months of age. Scale bar = 50 μm

Figure S3: Dextran uptake classification A) Lateral view of a 4 dpf zebrafish embryo immediately after being injected with a 10 kDa fluorescent dextran into the common-cardinal vein. B) Lateral view of a 4 dpf zebrafish embryo 2 hours after being injected with a 10 kDa fluorescent dextran into the common-cardinal vein. C) Three panels showing lateral views of fluorescent dextran uptake in the proximal pronephric tubule. Panel on left shows normal uptake, middle panel shows low uptake, and right panel shows no uptake.

Figure S4: NL-D3 proteinuria can be observed after a short incubation time Box and whisker plot showing the amount of NL-D3 present in the embryo medium of control and *lrp2a* morphants after 1 hour, 4 hours, 8 hours and 24 hours. Median is shown as a line and mean is shown as a red cross-hair.

Figure S5: Standard curve data A) Graph showing standard curve curated from data collected analysing 0.5 ng/ul to 0.0005 ng/ul. B) Close up visualisation of the graph in (A) in the region of the red-dotted box to highlight the lower concentrations of NL-D3 where most data points were collected.

Figure S6: TIDE analysis shows mutagenesis at gRNA site 1 in the Alport zebrafish crispants Histogram on the left shows the TIDE indel efficiency (calculated with the algorithm described on https://tide.nki.nl/) for three biological replicates induced by gRNA 1 for the col IV crispant zebrafish embryos. Histogram on the right shows the R_2 values for the corresponding indel efficiencies.

Figure S7: Single allele analysis of *col4a3* and *col4a4* crispants Sequences show wild type on top row with five sequencing analyses repeats of crispants below. *col4a3* sequences around the 4 gRNA sites are shown on the left, *col4a4* sequences around the 4 gRNA sites are shown on the right. Indels in individual alleles taken from mosaic animals were not detected in all four gRNAs. Only gRNA 4 in *col4a3* identified mutagenic alleles with two indels identified in all five clones analysed. In *col4a4*, gRNA1 and gRNA4 created indels.

Figure S8: Non-annotated TEM images of glomeruli in control and *col4a3* and *col4a4* crispants Panels show cross-section of glomerular filtration barrier under TEM. The pseudo-colouring from Figure 5 is shown as a separate image on the right. Podocyte foot processes (green), GBM (yellow), and endothelium (red) are highlighted. Scale bar = 500 nm

Figure S9: Increasing 2,3-BDM dose results in increasing effect on heart rate in zebrafish embryos Histogram shows heart rate (bpm) of wild-type embryos treated for 1 hour with vehicle (DMSO) or 2,3-BDM of varying concentrations highlighted.
